# Redox-Responsive Self-Assembled Amphiphilic Nanosheets from Polyglycerol Sulfate–Lipoic Acid Copolymers for Targeted Cancer Drug Delivery

**DOI:** 10.1021/acs.biomac.5c01204

**Published:** 2025-12-02

**Authors:** Taylor M. Page, Kai Ludwig, Muhammad Shayan Haider, Elisa Quaas, Alexandros Mavroskoufis, Peng Tang, Rui Chen, Jun Feng, Raju Bej, Katharina Achazi, Rainer Haag, Ievgen S. Donskyi

**Affiliations:** † Institut für Chemie Und Biochemie, 9166 Freie Universität Berlin, Takustr. 3, Berlin 14195, Germany; ‡ Forschungszentrum für Elektronenmikroskopie and Core Facility, 54203 BioSupraMol, Freie Universität Berlin, Fabeckstraße 36A, Berlin 14195, Germany; § Jyoti and Bhupat Mehta School of Health Sciences and Technology, 62397 Indian Institute of Technology Guwahati, Guwahati, Assam 781039, India

## Abstract

Targeted drug delivery systems that are stimuli-responsive offer great potential for enhancing the therapeutic activity of drugs, decreasing off-target effects, and improving bioavailability. This proof-of-concept study introduces an amphiphilic drug delivery system (DDS) capable of loading hydrophobic cargo. Elevated glutathione (GSH) levels, characteristic of certain types of cancer cells’ microenvironment, degrade the nanostructures and release the cargo. Linear polyglycerol sulfate (LPGS), known for its excellent biocompatibility, is combined with lipoic acid (LA). LA facilitates the formation of cross-linked nanosheet amphiphiles sensitive to reductive conditions. Morphological changes are observed by scanning electron microscopy (SEM), cryogenic transmission electron microscopy (Cryo-TEM), and cryogenic electron tomography (Cryo-ET) upon UV irradiation (*h*ν), creating a stable aggregate for loading hydrophobic cargo and assembling into sheets at elevated concentrations. The resulting material displays controlled release of model dyes under increased levels of GSH, tunable by the polymer size and LPGS:LA acid ratios. This behavior enhances targeted therapy and reduced off-target effects. Further loading with paclitaxel and subsequent release, together with *in vitro* assays, demonstrates the system’s compatibility with an anticancer drug.

## Graphical abstract

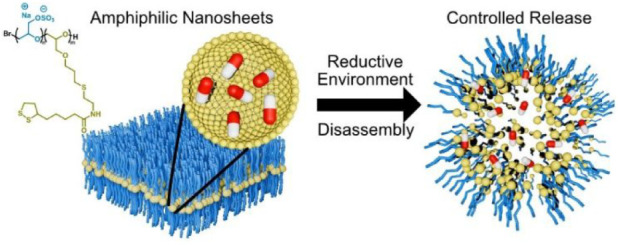

## Introduction

A major challenge in advancing DDSs is the development of stimuli-responsive carriers selective to tumor-specific conditions. Elevated reductive stress, a hallmark of many cancer tissues,^1,2^ including breast, ovarian, and lung cancer, exhibits elevated GSH levels^3,4^ due to rapid replication.^5^ Consequently, cancer cells exhibit an elevated mitochondrial reactive oxygen species (ROS). This leads to GSH concentrations up to 10 mM, 1000 times higher than in the extracellular matrix.^6,7^ Such conditions are ideal for triggered drug release induced by reductive stress.

Stimuli-responsive DDSs offer immense potential to improve targeted therapeutic treatment. By enabling controlled release, designed DDSs have the possibility to increase drug efficacy by mitigating exposure to healthy cells and plasma protein binding. They can also enhance a drug’s bioavailability over time while reducing side effects.^8^ Many chemotherapeutic drugs, such as doxorubicin, paclitaxel (PTX), and rapamycin, are minimally soluble in water, and thus induce side effects,^9^ due to poor targeting. Amphiphilic DDSs provide the benefit of being able to load and shield hydrophobic drugs, while remaining water-soluble.^10^

Polyglycerol (PG) and its derivatives have gained significant interest for DDS development due to their remarkable biocompatibility^11,12^ and cargo-loading potential.^13,14^ Polyglycerol sulfates have been investigated in stimuli-responsive materials^15,16^ due to their low cytotoxicity^17^ and low anticoagulant activity.^18^ Their surface charge helps to avoid renal elimination,^18^ while they also show high anticomplementary behavior^11,19^ and minimal protein binding, thereby avoiding side effects such as hemorrhage.^20^

Disulfide-containing motifs are often investigated in redox-responsive systems for reduction-responsive drug release.^21,22^ Lipoic acid (LA) contains a photoactive dithiolane, which has the ability to photo-cross-link polymers into networks^23^ and consequently provide sensitivity to reductive conditions.^24–27^ LA-based nanoparticles, formed through poly­(disulfide) cross-linking, have been investigated prior with polyethylene glycol (PEG)-based conjugates.^28–30^ However, PEG as a therapeutic is under mounting scrutiny due to the increasing prevalence of anti-PEG antibodies.^31,32^ Such a network of disulfide bonds creates a stable system under nonreductive environments. GSH, an antioxidant comprised of glycine, cysteine, and glutamic acid, is the most abundant low-molecular-weight thiol compound found in cells. It plays a key role in the cellular response to oxidants.^33,34^ Disulfide bonds, which are susceptible to GSH, have been used for stimuli responsiveness to stabilize carrier systems.^8^ Disulfide-containing polymers^35,36^ can degrade in high-GSH environments, promoting triggered release.^37^

In this work, we report the synthesis and characterization of a novel linear polyglycerol sulfate-*block*-lipoic amide (LPGS-*b*-LA) copolymer as a redox-responsive DDS. The LA moieties were photo-cross-linked, generating stable hydrophobic domains. Nile red (NR), a hydrophobic dye, was used as a model drug to evaluate the system’s loading capacity and release kinetics in response to elevated GSH levels. Four distinct polymers were developed, varying in block ratios and molecular weight to investigate how these parameters affect drug loading and release kinetics. The most promising candidate was further characterized by SEM, Cryo-TEM, and Cryo-ET, revealing tremendous changes in aggregation behavior. The material has nanoparticle morphology at low concentrations and sheetlike structures at higher concentrations. Cytotoxicity assays confirmed the biocompatibility of the material while confocal microscopy displayed efficient cellular uptake of NR-loaded carriers. The developed DDSs are stable in aqueous conditions and retain cargo until triggered release in reductive environments, where they steadily dissemble for sustained release. To assess therapeutic potential, paclitaxel, an FDA-approved tubulin-inhibiting anticancer drug since 1992,^37,38^ was loaded and quantitatively released during *in vitro* experiments on HeLa, MCF-7, and A549 cells. The final system demonstrated minimal leakage, selective GSH responsiveness, high loading capacity, and steady drug release. These features highlight its potential as an innovative DDS.

## Experimental Section

### Materials and Methods

All chemicals and solvents were obtained from commercial suppliers and used without further purification unless stated otherwise. Deionized water (DI water) was purified using a Millipore water purification system with a minimum resistivity of 18.0 MΩ·cm. Allyl glycidyl ether (AGE) was dried by stirring with CaH_2_, then distilled in vacuum before use and stored over molecular sieves. Glycidol (Sigma-Aldrich) was dried with CaH_2_, distilled before use, and stored at 4 °C. 2,3-Epoxypropan-1-ol (glycidol) was protected by reacting with ethyl vinyl ether to obtain ethoxy ethyl glycidyl ether (EEGE) according to a previously reported method.^38^ EEGE was further purified by stirring with CaH_2_, vacuum distilling, and storing over molecular sieves. Sulfamic acid (H_3_NSO_3_, Sigma-Aldrich), triethylamine (Et_3_N, Sigma-Aldrich), and 2-Hydroxy-4′-(2-hydroxyethoxy)-2-methylpropiophenone (TCI). All other reagents and solvents were purchased from different commercial suppliers and used as received, unless otherwise stated. Water was used from the Milli-Q Advantage A10 Water Purification System in all experiments. Regenerated cellulose dialysis tubes from Sigma-Aldrich (width: 32 mm, MWCO > 2000 g/mol) were used for purification of the synthesized compounds. Pur-A-Lyzer Midi dialysis kits with 3.5 kDa, for DDS_1_ and DDS_2_, and 6.0 kDa, for DDS_3_ and DDS_4_, molecular weights for DDS cutoffs were used.

### Elemental Analysis (EA)

Elemental analysis was performed by a Vario EL CHNS element analyzer using Elementar Analysensysteme GmbH (Langenselbold, Germany).

### Nuclear Magnetic Resonance (NMR)

All NMR spectra (^1^H) were recorded at 300 K on either a 600 MHz (JEOL Spectrometer ECZ600 S) or a 700 MHz (Bruker AVANCE700 spectrometer), as indicated. Chemical shifts (δ) were reported in parts per million, and the deuterated solvent peak was used as a standard.

### Fourier-Transform Infrared Spectroscopy (FTIR)

Fourier-transform infrared spectroscopy measurements were recorded using a PerkinElmer Spectrum Two FT-IR Spectrometer with a UATR Two accessory with a LiTaO_3_ detector.

### Zeta Surface (ζ) Potential

Zeta surface potential experiments were performed on a Malvern Zetasizer Ultra machine at 25 °C. Millipore water was used in all the experiments. Measurements were performed with a Malvern folded capillary zeta cell in automatic mode.

### Thermogravimetric Analysis (TGA)

Thermogravimetric analysis was conducted using a PerkinElmer TGA 8000, heated from 100 to 800 °C at 10 °C/min.

### Ultraviolet–Visible Spectroscopy (UV–vis)

UV–visible spectrum measurements were taken using an Agilent Cary 8454 and using a UV-Cuvette micro (70 μL). Blanks for each measurement were used, either PBS pH 7.4 or 10 mM GSH in PBS adjusted to pH 7.4 with 1.0 M NaOH. The lamp used for triggering the thiol–ene reaction was purchased from KESSIL (PR160L-390 nm, 40 W) in Taiwan.

### Gel Permeation Chromatography (GPC)

Gel Permeation Chromatography (GPC) measurements were performed using an Agilent 1100 solvent delivery system with a manual injector, isopump, and Agilent 1100 differential refractometer (Agilent Technologies, Santa Clara, CA, USA). Calibration standards were PSSS 210608 Puffer or Pullulan 210603 water, 1× with a pore size of 30 Å and 2× with a pore size of 1000 Å column, calibrated against Pullulan standards prior to measurements, depending on the charge or neutrality of the material. The Brookhaven BI-MwA7 angle light scattering detector was coupled with size exclusion chromatography (SEC) to measure the molecular weight of each fraction of the polymer that was eluted from the SEC columns.

### High-Performance Liquid Chromatography

Paclitaxel was quantified by HPLC (Nexera series System Shimadzu, consisting of Pump LC-40D XR, Degasser DGU-403, Injector LH-40, Column Oven CTO-40S, Detector SPD-M40), utilizing a Gemini 5 μm C18 100-Å column (Phenomenex) maintained at 22 °C. The solvent system used consisted of Water (A) and Acetonitrile (B) holding 50% B for a 20-min run time. Flow was maintained at a rate of 1 mL/min. Peaks were integrated at 227 nm.

### Cryo-Transmission Electron Microscopy

Perforated (1 μm hole diameter) carbon-film-covered microscopical 200 mesh grids (R1/4 batch of Quantifoil, MicroTools GmbH, Jena, Germany) were hydrophilized by a 60-s glow discharge at 8 W in a BALTEC MED 020 device (Leica Microsystems, Wetzlar, Germany). The samples of F-lPGS in water (1 mg/mL, 4 μL) were vitrified by automatic blotting and plunge freezing with an FEI Vitrobot Mark IV (Thermo Fisher Scientific Inc., Waltham, Massachusetts, USA) using liquid ethane as the cryogen. The vitrified specimens were transferred to the autoloader of an FEI TALOS ARCTICA electron microscope (Thermo Fisher Scientific Inc., Waltham, Massachusetts, USA). This microscope is equipped with a high-brightness field-emission gun (XFEG) operated at an acceleration voltage of 200 kV. Micrographs were acquired on an FEI Falcon 3 direct electron detector (Thermo Fisher Scientific Inc., Waltham, Massachusetts, USA) using a 100 μm objective aperture at a nominal magnification of 28 000×, corresponding to a calibrated pixel size of 3.69 Å/pixel.

### Synthesis of Linear Polyglycerol-*block*-poly­(allyl glycidyl ether) (LPG-*b*-PAGE)

The block copolymer LPG-*b*-PAGE was synthesized as previously published.^39,40^ In short, acetal-protected glycidyl (ethoxy ethyl glycidyl ether, EEGE) and allyl glycidyl ether (AGE) were copolymerized via ring-opening anionic polymerization using tetraoctylammonium bromide for initiation and triisobutyl aluminum as a catalyst in toluene. After 4 h of polymerizing EEGE, AGE was added to form discrete blocks. Following polymerization, the acetal was deprotected in acidic THF. The resulting product was then dialyzed against methanol for 3 days using regenerated cellulose (RC) Dialysis tubes with a molecular weight cutoff (MWCO) of 2 kDa. Four different polymers were synthesized, as outlined in Table 1, consisting of two 10 kDa and two 15 kDa polymers, each with large and small P­(AGE) segments. Average yield: 38.7%.

**Table 1:** Overview of Developed DDSs, with Regard to Their Degree of Sulfation, Amount of Lipoic Amide Modification, the Polymer’s Molecular Weight, and PDI^a^

DDS	Composition (%)	Sulfate (%)^b^	Lipoic Amide (%)^b^	MW (kDa)^c^	Dye Loading Content (%)	Dye Loading Efficiency (%)	*t*_1/2_ (hours)	*R* ^2^
1	LPGS_65_-*b*-LA_35_	65	35	10.2	17.0 ± 0.1	67.9 ± 0.4	16.2	0.957
2	LPGS_25_-*b*-LA_75_	25	75	10.6	29.6 ± 0.1	89.5 ± 1.5	58.1	0.926
3	LPGS_80_-*b*-LA_20_	80	20	17.9	19.4 ± 0.1	76.6 ± 0.2	61.9	0.931
4	LPGS_40_-*b*-LA_60_	40	60	14.7	39.4 ± 0.7	52.1 ± 1.0^d^	22.7	0.983

a Loading capacity, encapsulation efficiency, and release kinetics of NR encapsulated DDSs at 268 nm in 10.0 mM GSH over 7 days using two phase decay fitting. For corresponding graphs, see, Figures 2B, S17A-C.

b Percentages determined by NMR of LPGS-*b*-PAGE.

c Polymer MW represents LPGS-*b*-PAGE.

d DDS_1–3_ loaded with 30 wt % NR, and DDS_4_ loaded with 50 wt % NR.

### Synthesis of Linear Polyglycerol sulfate-*block*-poly­(allyl glycidyl ether) (LPGS-*b*-PAGE)

LPG-*b*-PAGE was sulfated following a previously published protocol.^41^ Briefly, LPG-*b*-PAGE (1.0 g, 0.19 mmol (7.6 mmol −OH, 1 −OH equiv) was dissolved in 60 mL of DMF, and sulfamic acid (1.48 g, 15.2 mmol, 2 −OH equiv) was dissolved in 40 mL of DMF and added dropwise. The reaction was stirred for 24 h. The product was purified by dialysis for 1 day against pH 11, 1.0 M NaCl, 1 day against 1 M NaCl, and 2 days against DI water, using RC Dialysis tubes with MWCO of 2 kDa. A dried sticky powder was obtained after lyophilization. Average yield: 42.7%.

### Synthesis of Linear Polyglycerol sulfate-*block*-cysteamine (LPGS-*b*-CA)

The allyl groups of LPGS-*b*-PAGE were coupled with cysteamine via a thiol–ene click reaction (1:5 molar equivalence of allyl groups to cysteamine) using UV (390 nm) light and 2-hydroxy-4′-(2-hydroxyethoxy)-2-methylpropiophenone (1.2 equiv) as a photoinitiator for 1 h in 50:50 water-to-ethanol, as per previously published literature.^40^ The product was dialyzed (2 days in 1 M NaCl, 3 days in DI water, RC Dialysis tubes with MWCO of 2 kDa) and dried via lyophilization. Average yield: 52.3%.

### Synthesis of Linear Polyglycerol sulfate-*block*-lipoic amide (LPGS-*b*-LA)

LPGS-*b*-CA (412 mg polymer, 65% by mass primary amine groups, 1.89 mmol, 1 equiv) was dissolved in minimal water (2 mL) and 100 μL TEA. Lipoic Amide–*N*-hydroxysuccinimide (LA-NHS, 687 mg, 2.27 mmol, 1.2 equiv) was then dissolved separately in 1.5 mL of DMF, combined with the aqueous polymer mixture, and allowed to stir overnight at room temperature. The resulting product was dialyzed against 1.0 M NaCl for 2 days, followed by DI water for 3 days. Average yield: 69.4%.

### Synthesis of Lipoic Acid–*N*,*N′*-Disuccinimidyl Carbonate (LA-NHS)

A modified synthesis from previously published work.^24^ Lipoic acid (5 g, 24.23 mmol, 1.0 equiv) and *N*,*N*′-Disuccinimidyl carbonate (7.45 g, 29.08 mmol, 1.2 equiv) were added to a round-bottom flask equipped with a stir bar and dissolved in acetonitrile (200 mL), rendering a yellow solution. Triethylamine was added (10.13 mL, 72.70 mmol, 3.1 equiv), and the reaction was stirred for 2 h under ambient conditions. The resulting solution was concentrated to approximately 50 mL by rotary evaporation, and the product was extracted with 275 mL of 5% NaHCO_3_. The product formed a pale-yellow precipitate, isolated by filtration, and dried under vacuum to afford LA-NHS. Yield: 83%, 10.33 g.

### DDS Preparation

LPGS-LA is dissolved in DI water, forming a solution of 10 mg/mL in a 10 mL round-bottom flask. The reaction mixture was then stirred and exposed to 370 nm light. After 1 h, the reaction was stopped, and the formed DDS was lyophilized.

### Cargo Loading of DDS

The loading cargo (NR or PXT) was dissolved in methanol and rotary evaporated in a 25 mL round-bottom flask, creating a thin film. Next, the DDS was dissolved to create a solution of 1.0 mg/mL in the flask. The mixture was stirred vigorously for 24 h. The product was filtered through a Sephadex G-25 filter and lyophilized.

### Release Studies

#### Nile Red

Nile Red-loaded DDS were taken and dissolved in an amount corresponding to 0.013–0.025 mg/mL (between 3 and 6 mg in 700 μL) of NR in 30 mL of solution to ensure that the maximum absorbance did not exceed 1.0. The DDS solution was placed in Pur-A-Lyzer Midi dialysis kits with a molecular weight cutoffs of 3.5 kDa, for DDS_1_ and DDS_2_, and 6.0 kDa, for DDS_3_ and DDS_4_. Then, the dialysis kits were placed in 50 mL Falcon tubes, filled with 30 mL of the appropriate solution (PBS at pH 7.4, 10 μM GSH in PBS at pH 7.4, or 10 mM GSH in PBS at pH 7.4), and placed in a shaking incubator at 37 °C. Absorbance measurements of the dialysis solvent were taken at various time intervals.

#### Paclitaxel

Paclitaxel-loaded DDS were taken and dissolved in a corresponding amount of 0.013–0.025 mg/mL (between 3 and 6 mg in 700 μL) of NR in 30 mL of solution, to ensure the maximum absorbance did not exceed 1.0. The DDS solution was placed in Pur-A-Lyzer Midi dialysis kits with molecular weight cutoffs of 3.5 kDa, for DDS_1_ and DDS_2_, and 6.0 kDa, for DDS_3_ and DDS_4_. Then, the dialysis kits were placed in 50 mL Falcon tubes, filled with 30 mL of the appropriate solution (PBS at pH 7.4, 10 μM GSH in PBS at pH 7.4, or 10 mM GSH in PBS at pH 7.4), and placed in a shaking incubator at 37 °C. Five mL aliquots of the dialysis solvent were taken at various time intervals, and the dialysis solution was replenished with fresh media. The aliquots were lyophilized, dissolved in 250 μL of acetonitrile, and their paclitaxel content was then measured by HPLC.

### Synthesis of Cy5-Maleimide

Cy5-Acid (24.0 mg, 42.6 μmol, 1.0 equiv) was dissolved in DMF (500 μL) at ambient temperature. HATU (21.1 mg, 55.4 μmol, 1.3 equiv) and Et_3_N (34.5 mg, 47.3 μL, 314 μmol, 8.0 equiv) were added subsequently, and the reaction mixture was stirred for 5 min. Then, amine (12.1 mg, 68.2 μmol, 1.6 equiv) was added, and the reaction was stirred overnight. After precipitation and decantation from Et_2_O (45 mL), the crude product was purified by automated flash chromatography (dry-loaded on Isolute, SiO_2_, DCM/MeOH 0% to 20%) and subsequent preparative HPLC (preparative column: Phenomenex Gemini-NX-C18, 5 μm, 250 × 30 mm, Solvent A: H_2_O/MeCN 95:5, solvent B: H_2_O/MeCN 5:95—10 min 100:0 isocratic, 11–30 min 70:30 isocratic). Analytical HPLC determined the purity of the collected fractions. Cy5-Maleimide (9.70 mg, 14.5 μmol) was obtained as a blue solid. Yield: 34%.

### Cy5-Malemide Conjugation

Cy5-malemide (1.0 mg, 0.003 mmol) was stirred with DDS_4_ in water (10 mg/mL, 1.0 mL) overnight. The product was filtered with Sephadex G-25 and provided for imaging. Yield: 46.4%.

### Cell Viability Study

Cytotoxicity of the polymer LPGS_40_-*b*-LA_60_ and nanoparticle DDS_4_ was studied using the cell viability assay Cell Counting Kit-8 (CCK-8) from Hycultec (HY-K0301) according to the manufacturer’s instructions. For the studies, HeLa cells (DSMZ no: ACC 57), A549 cells (DSMZ no: ACC 107), 16HBE14o-(HBE) cells (Millipore no: SCC150), and MCF-7 cells were cultured in DMEM (Dulbecco’s Modified Eagle Medium, 10569—DMEM, high glucose, GlutaMAX supplemented with l-Glutamine, 10 000 U mL^–1^ Penicillin-Streptomycin, and 10% Fetal Bovine Serum (all from Thermo Fisher Gibco BRL, Eggenstein, Germany). Cells were passaged every 3 to 4 days when reaching 70% to 90% confluency. For the assay, 90 μL of cells in medium were seeded in the inner wells of a 96-well plate (50 000 cells/mL) and incubated overnight at 37 °C and 5% CO_2_. In the outer wells, 90 μL of medium without cells was added for later background subtraction. On the next day, fresh dilutions of the compounds in sterile Milli-Q water were prepared, and 10 μL of each dilution was added to each of 3 wells containing 90 μL of preseeded cells in medium and 1 well with only 90 μL of medium to obtain the final test concentrations. Nontreated cells served as a control. After the addition of the compounds, the cells were incubated for another day before 10 μL of CCK-8 solution was added. After 3 h of incubation, absorbance was measured at a measurement wavelength of 450 nm and a reference wavelength of 650 nm with a Tecan plate reader (SPARK, Tecan Group Ltd., Männedorf, Switzerland). The whole experiment was repeated three times. The corrected absorbance (absorbance at 450 nm subtracted by the absorbance at 630 nm) was used to calculate the cell viability by first subtracting the background, setting the nontreated control to 100%, and then normalizing the values for the treated cells to the nontreated control. The mean with the standard deviation of all three assay runs was plotted for all compound concentrations.

### Cellular Uptake and Distribution

Cellular uptake and distribution of the Cy5-labeled or Nile Red-loaded DDS_4_ nanoparticles in HeLa cells were monitored by confocal laser scanning microscopy (CLSM). The cells were routinely cultivated in DMEM, as described above for the cytotoxicity study. For CLSM, 270 μL of cells in DMEM were seeded in each well of 8-well ibidi μ-slides (50 000 cells/mL). After 1 day, 30 μL of samples, 10× higher concentrated than the final test concentration, were added and incubated for 2 and 24 h. After 24 h, cell nuclei were stained with 1 μg/mL Hoechst 33342 (Life Technologies GmbH, Darmstadt, Germany) and, if needed, costained with LysoTracker Red (Thermo Fisher) according to the manufacturer’s instructions. Then, confocal images were taken by using an inverted confocal laser scanning microscope Leica DMI6000CSB SP8 (Leica, Wetzlar, Germany) with a 63x/1.4 HC PL APO CS2 oil immersion objective and LAS X software.

## Results

### Polymer Synthesis and Characterization

A collection of LPGS-*b*-LAs was created with varying LPGS-to-LA ratios to investigate the influence of the block composition on the encapsulation and release of carriers. Two molecular weight polymer chains (∼10 kDa and ∼15 kDa) were synthesized, each with a larger and a smaller LA core (see Table 1). The four diblock copolymers were synthesized by anionic ring-opening polymerization of ethoxy ethyl glycidyl ether (EEGE) and allyl glycidyl ether (AGE). Following deprotection, the LPG segment of the polymer was sulfated by using sulfamic acid.

Each step was confirmed with ^1^H NMR (Figure 1C; furthermore Figures S1-S4), elemental analysis (Table S3), FTIR (Figures S7-S10), and TGA (Figure S11). The sulfated polymer resulted in a negative shift in zeta surface (ζ) potential of the polymer from 37.2 ± 0.4 mV to −47.1 ± 3.4 mV. Next, a new FTIR signal appeared at 1210 cm^–1^ that corresponds to S=O stretching vibration. A significant increase in sulfur content was determined by elemental analysis, supporting upward of 90% sulfation. Further, according to TGA, all sulfated materials showed a significant change in the decomposition profile. This corresponds to a shift in decomposition of 60% for LPGS_60_-*b*-PAGE_40_ compared to its LPG_60_-*b*-PAGE_40_ precursor (Figure S11; further analysis can be found in the ESI).

**Figure 1:**
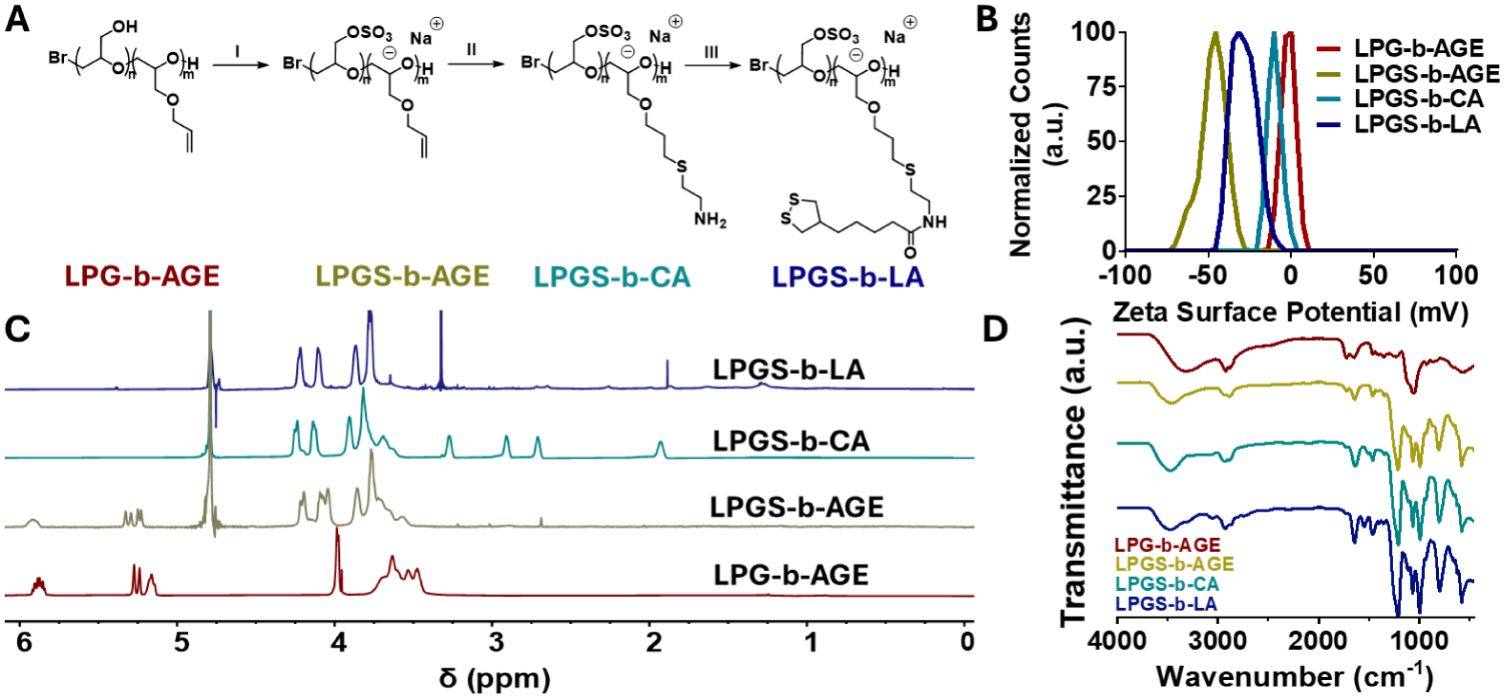
A) Synthetic pathway for preparing LPGS-Lipoic Amide Amphiphile LPGS_40_-*b*-LA_60_. I—Sulfamic acid, triethylamine, DMF, 24 h, 60 °C. II—Cysteamine HCl, 2-Hydroxy-4′-(2-hydroxyethoxy)-2-methylpropiophenone, water:ethanol 1:1, *h*ν, 1 h. III—Lipoic ester-NHS, DMF, 24 h. B) Zeta (ζ) surface potential for each synthetic intermediate. Measurements were taken at 1.0 mg/mL in deionized H_2_O. C) ^1^H NMR (600 MHz) spectra for each synthetic intermediate in various solvents. D) FTIR spectra of each synthetic step (further detailed spectra in ESI).

Next, the PAGE block underwent a thiol–ene click reaction with cysteamine, and the resulting primary amine reacted with lipoic acid *N*,*N*′-Disuccinimidyl carbonate (Figure 1A), according to a previously published protocol.^23^ The disappearance of alkene-related signals in the ^1^H NMR spectrum between 5 and 6 ppm confirmed the reaction. New signals appeared at 1.9, 2.8, 2.9, and 3.2 ppm. The ζ-potential shifted positively to −23.9 ± 0.8 mV. A slight decrease at 1720 cm^–1^ corresponding to a reduction in FTIR-active alkene stretches and an increase in C–S fingerprint stretches between 600 and 700 cm^–1^ further supported the conversion. The conjugation of lipoic acid is characterized 4-fold: First, we performed ^1^H NMR spectroscopy. Second, a shift to a more negative ζ-potential of −29.1 ± 0.5 mV supports the conversion of primary amines to lipoic amide. Third, by elemental analysis, the sulfur-to-carbon ratio indicated a conversion degree of 99%. Finally, the formation of the amide bond was confirmed by an FTIR signal at 1550 cm^–1^.

### DDS Formation, Characterization, and Cellular Uptake

To generate the DDSs, LPGS-*b*-LA amphiphiles were dissolved in DI water and cross-linked by 370 nm light irradiation for 1 h (Figure 2A). Prior to photo-cross-linking, the critical aggregation concentration (CAC) constant of LA LPGS_40_-*b*-LA_60_ was determined to be 0.28 ± 0.08 mg/mL (Figure S12). This relatively high value, and thus instability upon dilution, is attributed to the highly anionic LPGS shell of the aggregate.^42^

**Figure 2:**
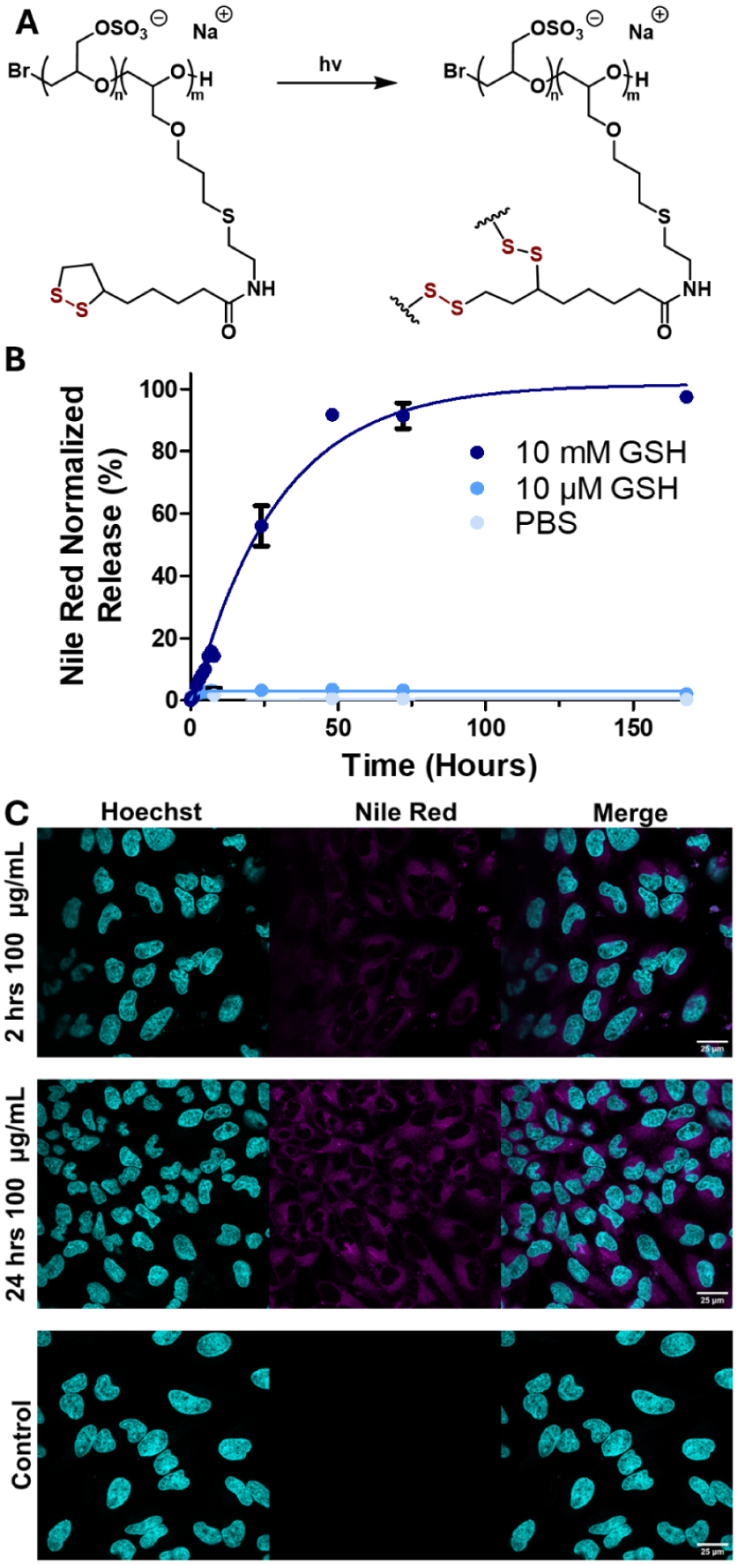
A) The light-mediated cross-linking through dithiolanes of LPGS-*b*-LA. B) Cumulative release of Nile Red-loaded DDS_4_ in PBS pH 7.4, PBS 10 μM GSH pH 7.4, and PBS 10 mM GSH pH 7.4. C) Confocal laser scanning microscope images of NR-encapsulated DDS_4_ cultured with HeLa cells at various times in DMEM (medium supplemented with 10% (v/v) FBS, 100 U/mL penicillin, and 100 μg/mL streptomycin.) Nile Red is shown in magenta, and the nuclei stained with Hoechst 4432 are in cyan.

By design, lipoic acid presents as a hydrophobic motif that readily undergoes light-initiated polymerization by homolytic cleavage.^43^ This process forms a cross-linked core after 1 h of irradiation. The light-triggered network formation utilized the dithiolanes present in the LA to form a network of adjacently joined dithiols. UV–vis absorption spectra support successful dithiolane ring opening with apparent photobleaching (Figure S14), concurrent with previously reported analogous findings using thioctic acid.^24^ Formed disulfide bonds are generally stable in biological systems, as noted by protein structures that are often dependent on disulfide bridges for their tertiary and quaternary structures,^44^ and consequently, their function.

NR was chosen as a model dye due to its hydrophobicity, a similar obstacle faced by anticancer drugs when administered. The DDSs were loaded by dissolving NR in methanol and then removing the solvent by rotary evaporation, followed by high vacuum, creating a thin film. Respective DDSs, suspended in DI water at 10 mg/mL, were added to the flask and stirred vigorously for 24 h. The resulting encapsulated DDSs were purified by size exclusion chromatography using Sephadex G-25, with DI water as an eluent, to exclude any unencapsulated dye. The loaded DDSs were then dried via lyophilization. The loading capacity and encapsulation efficiency were determined by UV–vis against an NR standard calibration. The loading capacity increases with both the percentage of LA-modified polymer and the size of the polymer used to create the DDSs. For the 10 kDa DDS_1_ and DDS_2_, the percentage of LA is almost doubled from 35% to 75%, respectively, while the loading capacity follows from 17% to 30%. Similarly, for DDS_3_ and DDS_4_, the percentage of LA is 3-fold larger, from 20% to 60%, resulting in loading capacities from 26% to 39%.

Since the premise of the developed DDSs depends on elevated GSH levels in cancer cells to selectively trigger disulfide decomposition, the DDSs’ stability and consequent releasing capabilities were assessed using 10 mM GSH. Since GSH contains a free thiol, we expect it to undergo thiol–disulfide exchange with the poly­(disulfide) core, where the free thiol nucleophilic attacks the core disulfide bonds, degrading the core. The DDS’s sensitivity to GSH-targeted release is influenced by the polymer length and composition used to form the DDS nanoparticles. Over a period of 7 days, the release was followed by UV–vis spectroscopy to quantify the amount of NR released from the DDS nanoparticles. In GSH-free PBS, the DDSs remained stable, largely retaining NR, with less than 15% release for all DDSs (Figures 2B, S15A-C), even after 7 days. This also supports that the dye is held in the core and is not bound by electrostatic interaction with anionic LPGS. The stability of the DDSs in PBS is as expected across all four DDSs. The presence of 10 mM GSH begins to degrade the poly­(disulfide)-stabilized core within 1 h.

Each DDS presents a unique degradation half-life (*t*_1/2_) (Table 1, Figure S17). Larger LPGS domains can play two roles: hindering the diffusion of GSH through its ionic shell and promoting nanoparticle disintegration due to hydrophobic effects. Conversely, the increasing size of the LA core likely preserves the integrity of the nanoparticle by requiring more GSH and time to render the structure unstable, while the structure is preserved longer by hydrophobic effects. DDS_1_ degrades the quickest, with a *t*_1/2_ of 16.2 h, which is tipped toward rapid degradation by a smaller LA core, and less shielding from the ionic LPGS shell. DDS_2_ presents a larger *t*_1/2_, due to a larger core, and likely experiences prolonged hydrophobic aggregation, necessitating more incubation time with GSH to degrade the core further to render the system dynamic enough for controlled release. The larger DDSs, DDS_3_ and DDS_4_ appear to be largely controlled by the size of the ionic LPGS shell, where DDS_3_ has the longest *t*_1/2_ of 61.9 h and 80% LPGS. Each DDS offers different advantages, depending on the desired sustained release profile or more rapid delivery of cargo under reductive conditions. DDS_4_ was selected as the best candidate for further investigation due to its large loading capacity. Consequently, it has the best potential to increase the therapeutic index of a given amount of drug loaded by preventing a rapid spike in free drug associated with intravenous injections, thus lowering the toxicity and side effects. The resulting cross-linked poly­(disulfide)-containing DDS_4_ exhibited different morphological properties than the amphiphilic precursor LPGS_40_-*b*-LA_60_.

Cryo-TEM, Cryo-ET, and SEM of DDS_4_ at varying concentrations revealed a concentration-dependent morphology. At low concentrations (<0.1 μg/mL), SEM displayed predominantly spherical nanoparticles, approximately 75 nm (Figures 3A,B, S15A,B). In contrast, at higher concentrations (>1.0 μg/mL), extended lamellar or sheet-like assemblies were observed (Figures 3 E,F and S15C–F), and additionally confirmed with Cryo-TEM (Figure 3G) and Cryo-ET (Figures 3H,IJ,K, S16A-B, Supporting Video) to avoid fixation or drying artifacts. The observed concentration-dependent morphology arises from the amphiphilic balance between the hydrophilic LPGS block and the hydrophobic, photo-cross-linked lipoic amide domains. At concentrations up to 1 μg/mL, the polymers form small, spherical nanoparticles stabilized by the ionic LPGS shell. As the concentration increases, enhanced hydrophobic interactions between the cross-linked LA domains promote lateral aggregation, leading to the formation of extended lamellar or sheet-like structures, as confirmed by SEM and Cryo-TEM. Cryo-TEM and Cryo-ET images were performed at 10 and 5 mg/mL, respectively. These concentrations were required because the methods have limited sensitivity at lower sample amounts. At further dilution, imaging becomes difficult due to poor contrast and a low signal-to-noise ratio. This self-assembly is consistent with previous reports of polyglycerol-based amphiphiles forming supramolecular structures, driven by hydrophobic interactions.^45^ These findings support that DDS_4_ forms stable, cross-linked nanoparticles in aqueous solution, capable of adopting sheet-like morphologies at higher concentrations.

**Figure 3:**
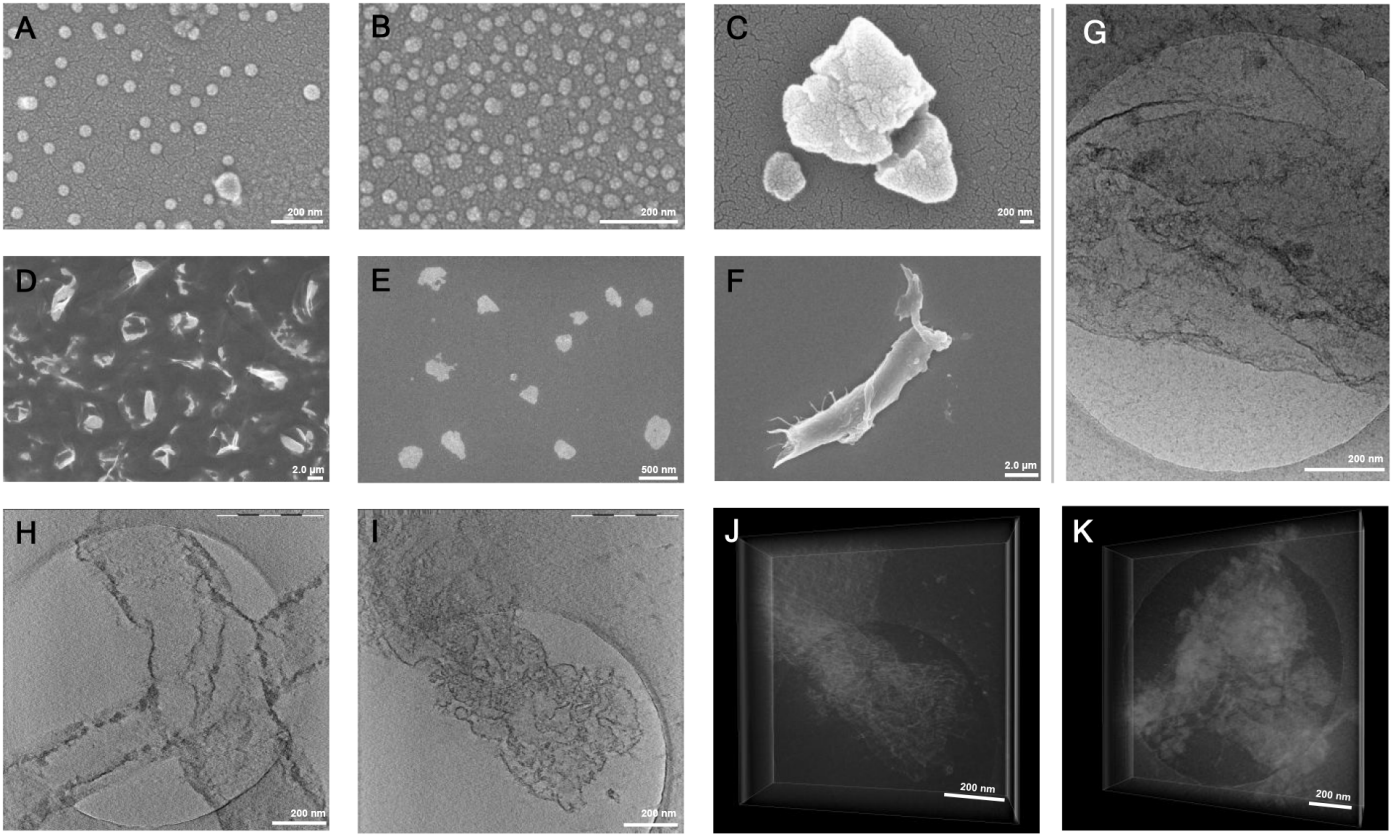
SEM and Cryo-TEM images of DDS_4_. SEM images of samples correspond to different concentrations prior to drop casting, gold coating, and imaging: A) 1.0 ng/mL, B) 10 ng/mL, C) 0.1 μg/mL, D) 1.0 μg/mL, E) 10 μg/mL, and F) 0.1 mg/mL. G) Cryo-TEM image of DDS_4_ vitrified at 5.0 mg/mL. H–K) 3D volumes, reconstructed from tomographic tilt series (±64°, 2° increment) of DDS_4_, embedded in amorphous ice at 5.0 mg/mL: H,I) 2D slices through the 3D volume; J,K) Voltex presentation of the complete 3D volume (note the inverted contrast).

### Mechanism of Uptake

Following the analysis of redox-triggered controlled release, we investigated the cellular distribution and uptake behavior of DDS_4_. To evaluate its ability to transport hydrophobic cargo into cancer cells, NR-loaded DDS_4_ was incubated with HeLa cells at 50 μg/mL for 2 and 24 h. Additional analysis of the release profile in PBS with GSH (10 μg/mL) at pH 7.4 for DDS_4_ demonstrated minimal premature leakage in conditions consistent with the reductive conditions of the extracellular matrix (Figure 2B). As shown in Figure 4, fluorescence microscopy revealed a strong distribution of NR selectively within the cytoplasm by the 2-h mark. After 24 h, the NR fluorescence increased, indicating efficient and progressive intracellular accumulation. Given the anionic surface charge of LPGS, endocytosis is the most likely entry route, mediated by interactions with positively charged membrane proteins.^46^ The dotted intracellular NR fluorescence pattern observed is consistent with endosomal localization, analogous to previous findings with doxorubicin-loaded micellar DDSs.^47,48^ To support the endosomal trafficking, Cy5-labeled DDS_4_ was synthesized through maleimide–thiol coupling and purified through size exclusion chromatography (Sephadex G-25). Cy5-DDS_4_ was then incubated with HeLa cells for 4 and 24 h, alongside LysoTracker Red (L7528, Thermo Fisher). From Figure 4, Cy5 fluorescence was seen within cells after 4 h of incubation, and colocalization with LysoTracker was evident by 24 h. Further assays were conducted at 100 μg/mL, which are consistent in terms of uptake. However, the samples tended to form sheets that cannot be readily absorbed due to their observed aggregation (see Figure S18). An equilibrium is likely to exist between the bigger sheets and the smaller particles that can then be taken up by cells. The smallest sheet components, which are around 75 nm in size when dried (according to SEM, Figure 3A,B), were consistently detected by DLS across dilutions from 10 mg/mL to 5 μg/mL (Figure S13). Remarkably, polymer-bound Cy5 dye penetrates inside cells as rapidly as within 4 h of incubation. These observations suggest that DDS internalization proceeds primarily through endocytic vesicles and subsequently undergoes lysosomal compartmentalization. Therefore, we postulate that the DDS is primarily internalized prior to disassembly, enabled by a rapid cellular uptake mechanism, and minimal leakage in extracellular matrix-like conditions, as previously demonstrated.^49^ Notably, intracellular localization was observed with both NR-loaded DDS_4_ and Cy5-DDS_4_. These results support that the DDS effectively penetrates cell membranes and delivers cargo intracellularly while maintaining its poly­(disulfide) cross-linked core integrity due to the prolonged half-lives prior to disassembly.

**Figure 4:**
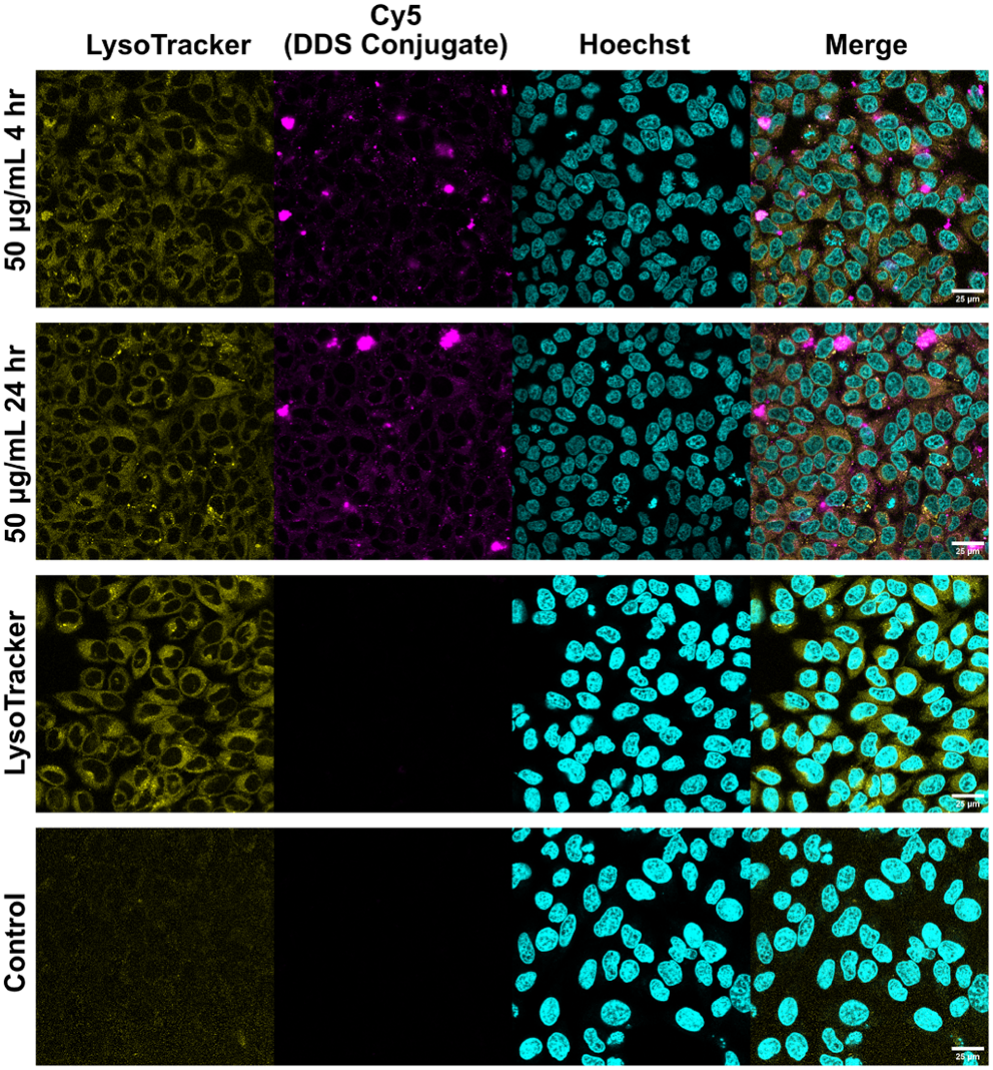
Confocal laser scanning microscope images of HeLa cells cultured for various times and concentrations of DDS_4_ with a covalently bound maleimide-modified Cy5 dye and LysoTracker. Cy5 is shown in magenta, Lysotracker is shown in yellow, and the nuclei stained with Hoechst 44432 are in cyan. Scale bar = 25 μm.

### Cytotoxicity

To assess the biocompatibility potential of the developed DDS system, cell viability assays were conducted on three different cell lines, HeLa, HBE (16HBE14o-), and A549 cells. Cells were treated with DDS_4_ and its precursor (LPGS_40_-*b*-LA_60_) at concentrations up to 100 μg/mL using a Cell Counting Kit-8 (Figure 5A,B). Given that the outer shell of the DDSs is LPGS, it was expected that the material would exhibit minimal cytotoxicity, which is consistent with our findings. All three cell lines proliferated in the presence of both DDS_4_ and its precursor. Yet, a slight reduction in viability at the highest concentration of DDS_4_ was observed. This may be attributed to residual free thiols generated during photo-cross-linking.^50,51^ Overall, the low toxicity profile supports the potential biocompatibility of the developed system.

**Figure 5:**
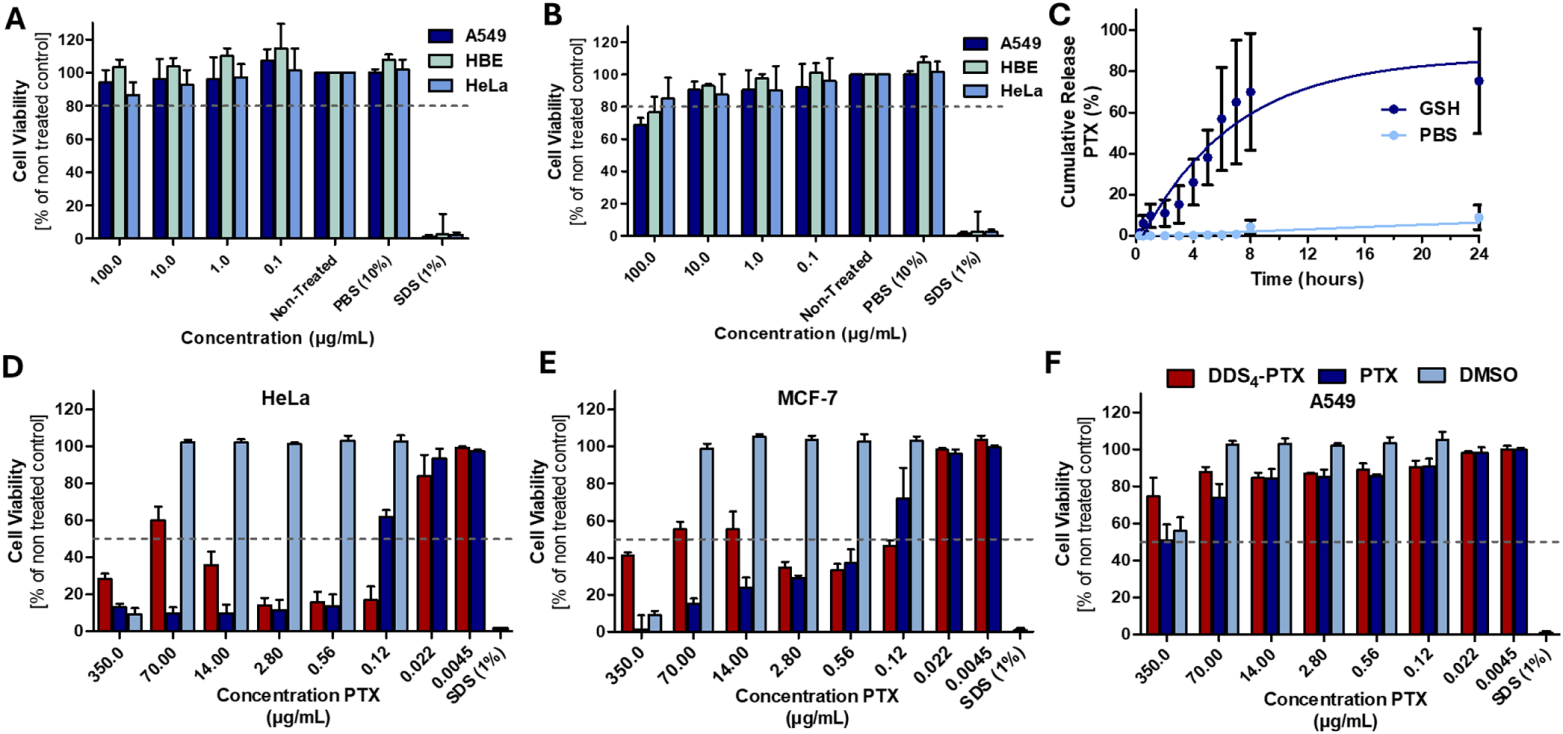
In vitro CCK8 cell viability assay of cells treated with A) LPGS_40_-*b*-LA_60_ and B) DDS_4_ formed on A549, HBE, and HeLa cells from 100 to 0.1 μg/mL as a percentage of the nontreated control. C) Release study of PTX-encapsulated DDS_4_ over 24 h in 10 mM GSH pH 7.4 (*n* = 3) and a PBS pH 7.4-only control (*n* = 3), monitored by HPLC. The data were fitted to a one-phase decay model using GraphPad Prism. In vitro CCK8 cell viability assay of cells treated with DDS_4_ loaded with PTX, PTX dissolved in DMSO, and DMSO on D) HeLa cells, E) MCF-7 cells, and F) A549 cells. Note that the concentration displayed corresponds to 34.3% encapsulation of PTX in DDS_4_, and the concentration of DMSO corresponds to the amount of DMSO present in PTX conditions.

### Paclitaxel Loading and Controlled Release

To evaluate the therapeutic potential of the developed DDSs, paclitaxel was encapsulated into DDS_4_ and assessed for redox-responsive release. A considerable loading capacity of 34.3 ± 19.2% and an encapsulation efficiency of 114.4 ± 64.1% were achieved for paclitaxel in DDS_4_, demonstrating the carrier’s encapsulation effectiveness.

Drug release was monitored under simulated reductive conditions: 10 mM GSH in PBS (pH 7.4) and GSH-free PBS. Aliquots were removed, processed for high-performance liquid chromatography (HPLC) analysis, and replenished with fresh buffer to maintain consistent conditions. The release profile followed an exponential release and was fitted to a one-phase decay. After 24 h, approximately 80% cumulative release was observed, with a *t*_1/2_ of 4.6 h. In contrast, the GSH-free control exhibited negligible leakage, even after 7 days.

To investigate the effect of PTX-loaded DDS_4_, viability assays were conducted on three well-established cancer cell lines: HeLa, MCF-7, and A549 (Figure 5D-F). PTX-loaded DDS_4_ was compared to free PTX dissolved in dimethyl sulfoxide (DMSO), and DMSO was used as a control. HeLa and MCF-7 cell lines indicated that concentration, and in turn morphology, is a contributing factor to DDS uptake and cell viability. The concentration of PTX in DMSO corresponded to the concentration of PTX loaded into DDS_4_. At DDS_4_ concentrations between 1.6 and 0.2 mg/mL, DDS-PTX incubated on HeLa and MCF-7 cells maintained higher viability than those treated with PTX. These observations demonstrate that cellular uptake above 1.0 μg/mL is accelerated due to the persistence of sheets, as previously observed in SEM images. The most effective concentration appears to be 1.6 μg/mL DDS_4_–PTX (0.56 μg/mL PTX) for MCF-7 and 0.8 μg/mL (0.28 μg/mL PTX) for HeLa. These represent the highest concentrations where PTX-loaded DDS_4_ exhibits viability analogous to that of free PTX (Figure 5D,E). At these concentrations, SEM analysis indicates that DDS_4_ forms smaller nanostructures, which are more favorable for cellular uptake compared to sheet-like structures. This correlates with the concentration range that supports efficient drug delivery and reduced cell viability. In contrast, A549 cells that have higher intrinsic microtubule dynamicity and susceptibility to clear PTX^52,53^ exhibited reduced toxicity to both DDS_4_ and free PTX (Figure 5F). These results support DDS’s potential as a redox-responsive carrier for targeted cancer therapy with selective release characteristics.

## Conclusions

In this study, we introduce a novel class of redox-responsive drug delivery systems based on linear polyglycerol sulfate-*block*-lipoic amide amphiphiles, which assemble into nanosheets. This proof-of-concept investigation highlights the utility of LA as a photo-cross-linked and redox-sensitive motif capable of forming stable, core cross-linked carriers, capable of encapsulating hydrophobic cargo. In reductive conditions prevalent in tumor microenvironments, formed poly­(disulfide) bonds selectively degrade, resulting in a controlled and sustained release of NR. The release kinetics were found to be tunable based on the ratio of anionic LPGS to the hydrophobic LA core, with *t*_1/2_ ranging from 16 to 62 h. Among the four formulations examined, DDS_4_ emerged as the lead candidate, combining a high drug-loading capacity with *t*_1/2_ near 24 h. Microscopy indicates a concentration-dependent morphological shift from discrete nanoparticles, as observed in SEM, to sheet-like structures above 1.0 μg/mL, supported by further SEM images, Cryo-TEM, and tomography. Confocal microscopy studies demonstrated rapid cellular uptake within 2 h via endosomal pathways. Importantly, the system displayed minimal toxicity across multiple cell lines and retained therapeutic activity when loaded with PTX, demonstrating comparable performance to the drug alone at 1.6 μg/mL of DDS_4_–PTX (0.56 μg/mL PTX) for MCF-7 and 0.8 μg/mL (0.28 μg/mL PTX) for HeLa, but with the advantages of redox-triggered release. Overall, the LPGS-*b*-LA platform presents a promising strategy for tailored, redox-responsive drug delivery, offering low cytotoxicity, structural stability, minimal off-target effects induced by the drug, and premature leakage, as well as controlled release.

## Supplementary Material





## Notes

Additional characterization and analysis are available: ^1^H NMR spectra of synthesized materials; Mass Spectrum of NHS–Lipoic acid; FTIR, TGA, Zeta surface potential, and elemental analysis of each developed DDS polymers and their precursors; GPC of each synthesized polymer and its LPGS-AGE form; DLS analysis of DDS4 and its polymeric precursor LPGS40-*b*-LA60; UV–vis spectra of formed DDS4; Additional SEM and Cryo-electronic tomography images of DDS4; Release data for each DDS_1_–DDS_3_ loaded with Nile red, and release data for DDS_4_ loaded with paclitaxel over 72 h; Additional confocal laser scanning microscope images of Cy5-DDS_4_ at 100 μg/mL; Scheme for Cy5 preparation and ^1^H NMR spectrum, ^13^C NMR spectrum, and mass spectrum of Cy5; Additional figures, tables, experimental details, and data supporting the findings of this study (PDF)
Cryo-ET_3D-volume (MP4)

## Abbreviations

GSH: glutathione
LPG: linear poly glycerol
LPGS: linear polyglycerol sulfate
ROS: reactive oxygen species
CA: cysteamine
LA: lipoic acid
EEGE: ethoxy ethyl glycidyl ether
AGE: allyl glycidyl ether
THF: tetrahydrofuran
DI: deionized
RC: regenerated cellulose
GPC: gas phase chromatography
NMR: nuclear magnetic resonance
FTIR: Fourier-transform infrared spectroscopy
TGA: thermogravimetric analysis
NR: Nile red
PTX: paclitaxel
PBS: PBSphosphate-buffered saline
GSH: glutathione
SEM: scanning electron microscopy
Cryo-TEM: cryogenic transmission electron microscopy
Cryo-ET: cryogenic electron tomography
HPLC: high-performance liquid chromatography
