# Supplementary material for: Redox-Responsive Self-Assembled Amphiphilic Nanosheets from Polyglycerol Sulfate–Lipoic Acid Copolymers for Targeted Cancer Drug Delivery
Source: Biomacromolecules. 2025 Dec 2;27(1):249–58. doi: 10.1021/acs.biomac.5c01204 (PMC12801293; doi:10.1021/acs.biomac.5c01204)
Supplement: Supplementary file 1 [file bm5c01204_si_001.pdf]

Supporting Information

Redox-Responsive Self-assembled Amphiphilic Nanosheets of Polyglycerol Sulfate - Lipoic Acid Copolymers for Targeted Cancer Drug Delivery

Taylor M. Page [a], Kai Ludwig [b], Muhammad S. Haider[a], Elisa Quaas[a], Alexandros Mavroskoufis [a], Peng Tang [a], Rui Chen [a], Jun Feng, Raju Bej,[c] Katharina Achazi [a], Rainer Haag [a], Ievgen S. Donskyi [a]**

T. M. Page, M. S. Haider, E. Quass, A. Mavroskoufis, P. Tang, R. Chen, J. Feng, K. Achazi, R. Haag, I. S. Donskyi

[a] Institut für Chemie und Biochemie, Freie Universität Berlin, Takustr. 3, 14195 Berlin, Germany

K. Ludwig

[b] Forschungszentrum für Elektronenmikroskopie and Core Facility BioSupraMol Freie Universität Berlin Fabeckstraße 36A, 14195 Berlin, Germany

R. Bej

[c] Jyoti and Bhupat Mehta School of Health Sciences and Technology, Indian Institute of Technology Guwahati, Assam-781039, India

*Email: haag@zedat.fu-berlin.de, edonskoy89@zedat.fu-berlin.de

Contents

^{1}H Nuclear Magnetic Resonance 3
Mass Spectrum 8
Fourier Transform Infrared Spectroscopy 9
Thermogravimetric Analysis 13
Zeta Surface Potential 14
GPC 14
Elemental Analysis 15
Critical Aggregation Constant 16
Photocrosslinking 17
Scanning Electron Microscope Images 18
Cryo-Electron Tomography 18
Release Data 19
Cy5 Dye Preparation 21

^{1}H Nuclear Magnetic Resonance

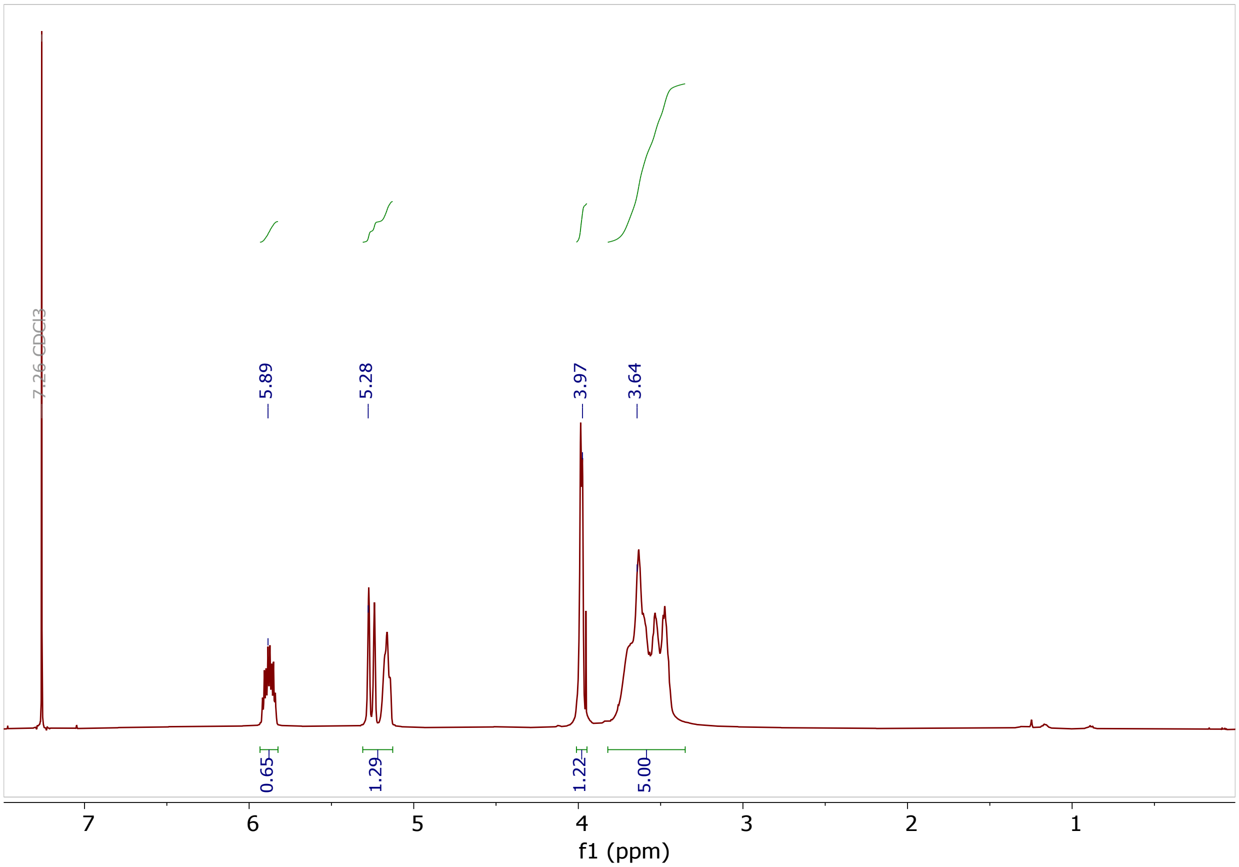

Figure S1. ^{1}H NMR in $CDCl_3$ of LPG_{40}-b-AGE_{60}, DDS_4 precursor. ^{1}H NMR ($CDCl_3$, 600 MHz) LPG-b-PAGE: Repeating units (n-LPG, m-PAGE) δ = 3.4–3.8 ppm (polymer backbone: C**H2**–C**H**(C**H2**O)O, n + m 5H), 3.95-4.05 ppm (O–C**H2**–CH═CH2, m 2H), 5.10–5.30 ppm (C**H2**═CH–, m 2H), 5.80–5.95 ppm (CH2═C**H**–O), m 1H)

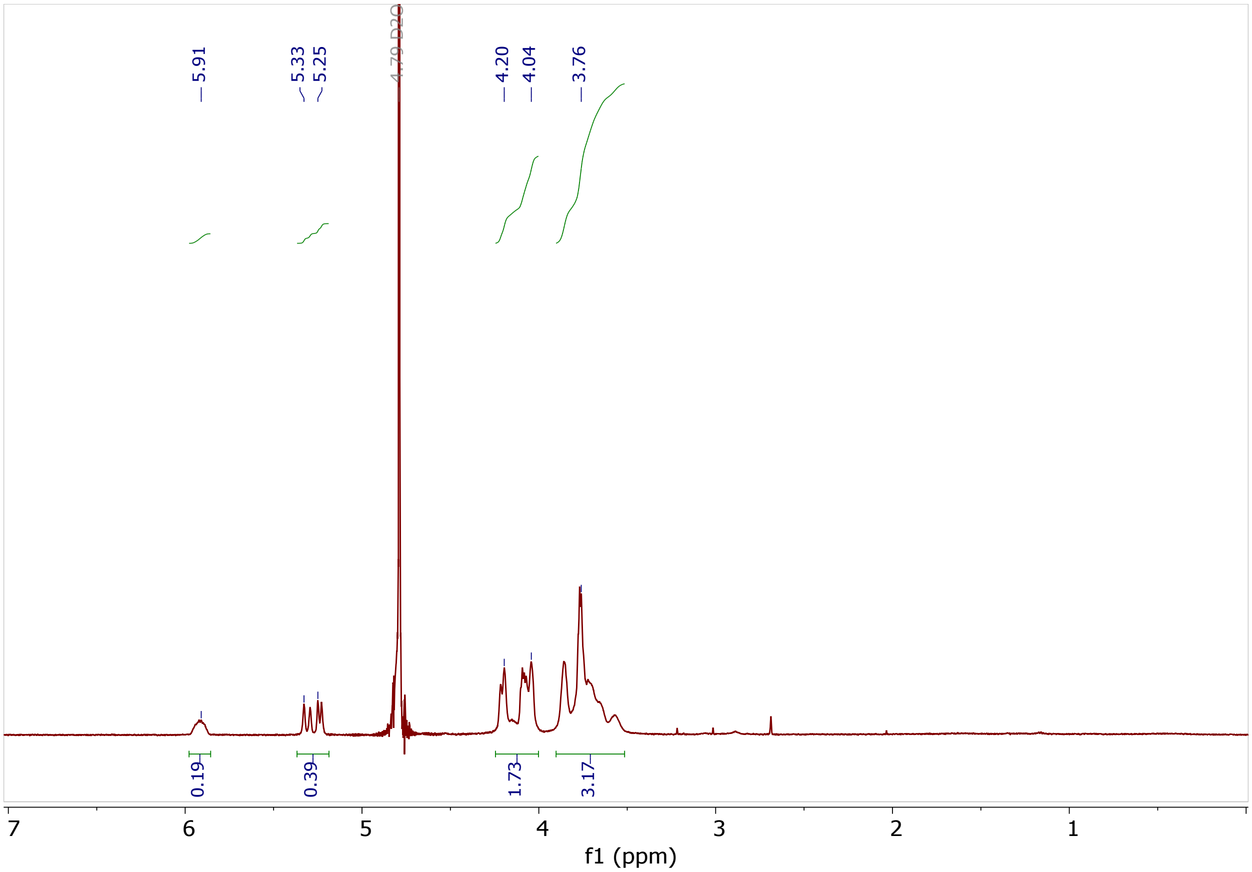

Figure S2. ^{1}H NMR in D_2O of $LPGS_{40}$-b-AGE_{60}. ^{1}H NMR (D_2O, 600 MHz): δ = 3.50–3.90 ppm (polymer backbone: C**H2**–C**H**(C**H2**O) O, n 5H), (C**H**2−C**H**−C**H**2−OSO3Na, m 5H), 3.95-4.05 ppm (O–C**H2**–CH═CH2, m 2H), 5.20–5.35 ppm (C**H2**═CH–, m 2H), 5.85–6.00 ppm (CH2═C**H**–, m 1H).

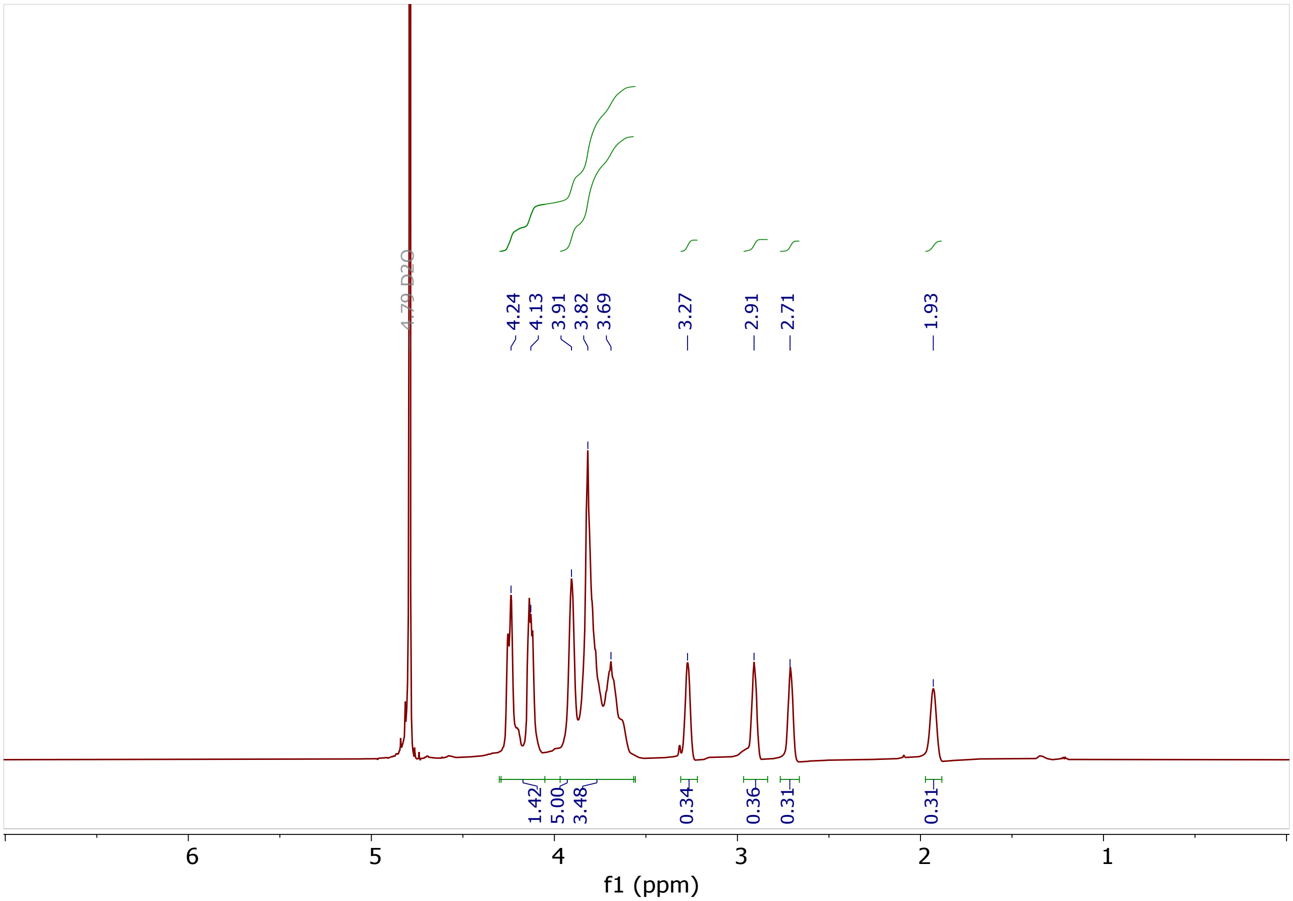

Figure S3. ^{1}H NMR in D_2O of LPGS-CA, DDS_4 precursor. ^{1}H NMR (D_2O, 600 MHz): δ = 1.9 ppm (-N**H₂**, m 2H), 2.8 ppm (-C**H₂**-S-CH₂-, m 2H), 2.9 ppm (-CH₂-S-C**H₂**-, m 2H), 3.2 ppm (-C**H₂**-NH₂, m 2H), 3.50–3.90 ppm (polymer backbone: C**H2**–C**H**(C**H2**O)O, n 5H), (C**H**2−C**H**−C**H**2−OSO3Na, n 5H), 3.95-4.05 ppm (O–C**H2**–CH═CH2, m 2H).

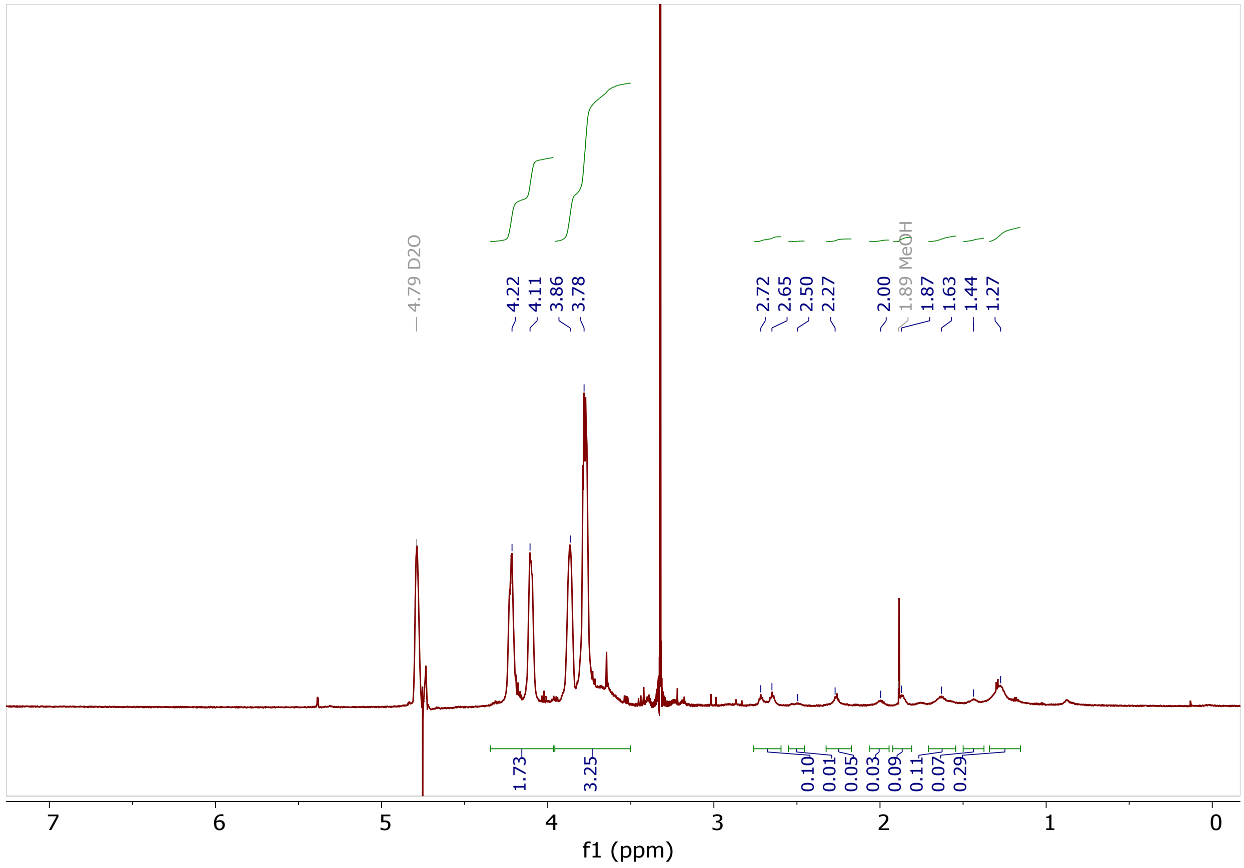

Figure S4. ^{1}H NMR in D_2O of LPGS-b-LA, DDS_4 precursor. ^{1}H NMR (D_2O, 700 MHz): δ = 0.88 ppm (-CH-C**H**$_2$-CH$_2$, m 1H), 1.27 ppm (-CH-C**H**$_2$-C**H$_2$**-C**H$_2$**-CH$_2$, m 5H), 1.63 ppm (-S-C**H$_2$**-CH$_2$-NH-, m 2 H), 1.87 ppm (-S-CH$_2$-C**H$_2$**-NH-, m 2H), 2.00 ppm (S-CH$_2$-C**H**$_2$-CH-S,-CH$_2$, m 1H), 2.27 ppm (S-CH$_2$-C**H**$_2$-CH-S,-CH$_2$, m 1H), 2.60-2.75 ppm (-S-C**H$_2$**-CH$_2$-NH-, and -CH$_2$-C**H**-CH$_2$,-S- m 3H), fi2.65 ppm (-CH$_2$-C**H**$_2$-CONH-, m 2H), 3.50–3.90 ppm (polymer backbone: C**H2**–C**H**(C**H2**O)O, n 5H), (C**H**2−C**H**−C**H**2−OSO3Na, n 5H), 3.95-4.05 ppm (O–C**H2**–CH═CH2, m 2H).

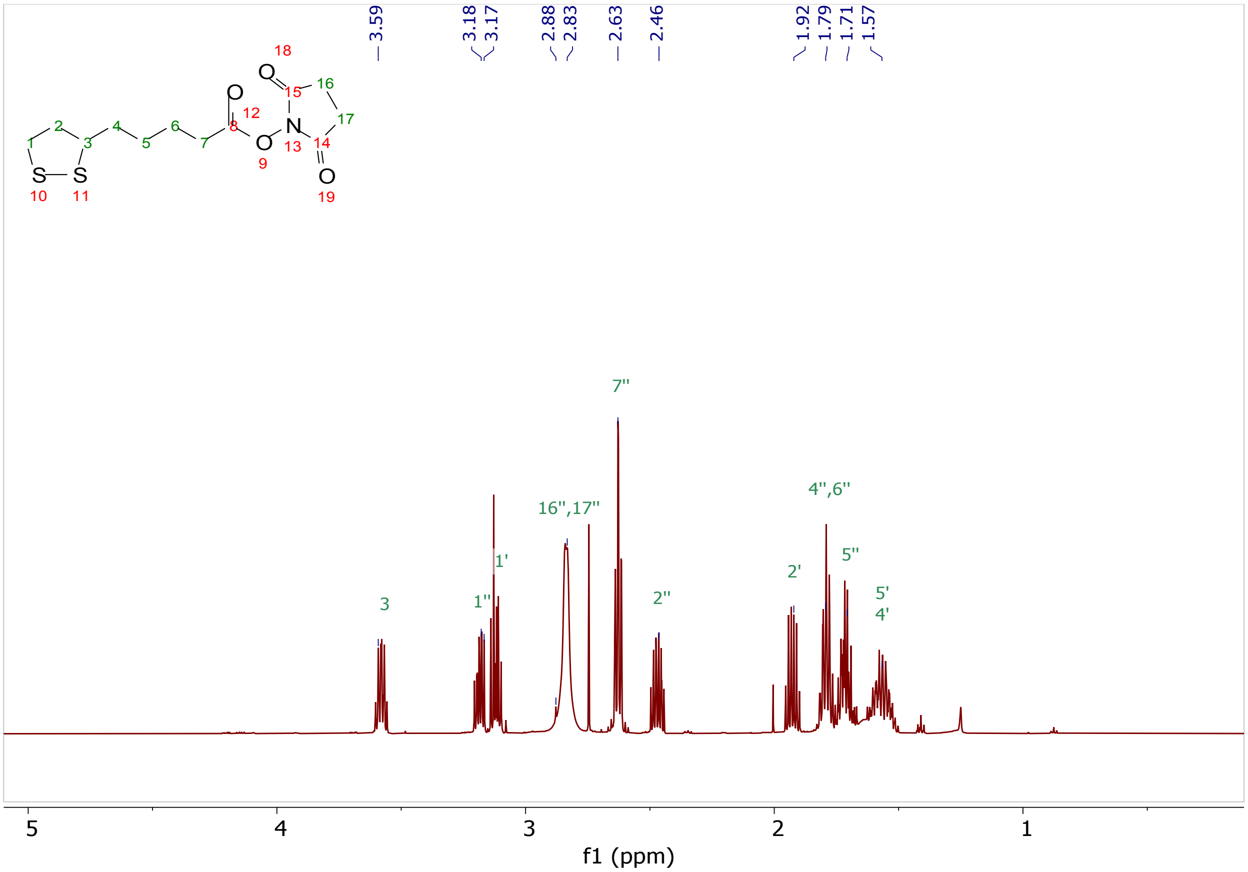

Figure S5. ^{1}H NMR *(600 MHz)* of Lipoic Acid - N, N'-Disuccinimidyl carbonate (LA-NHS) in $CDCl_3$. δ = 1.57 ppm (-CH_2-C**H**$_2$-C**H**$_2$- CH_2-, m 2H), 1.71 ppm (-CH_2-C**H**$_2$-CH_2- CH_2-, m 1 H). 1.75 (--CH-C**H**$_2$-CH_2-C**H₂**- CH_2-, m 3H), 1.92 ppm (-S-CH_2-C**H**$_2$-CH-S,-CH_2-, sx 1H), 2.46 ppm ((-S-CH_2-C**H**$_2$-CH-S,-CH_2-, sx 1H), 2.63 ppm (-CH_2-C**H₂**-COO-, dt 2H), 2.83 (-CO-C**H₂**-C**H₂**-CO-, m 4Hz) 3.1-3.2 ppm (-S-C**H₂**-CH_2-, m 2H), 3.58 ppm (-CH_2-C**H**-S,-CH_2, q 1H) (See Figure S5). HRMS (ESI, pos.): *m/z* calc. for $C_{12}H_{17}NO_4S_2$ [M]: 303.06, found 303.0576.

Mass Spectrum

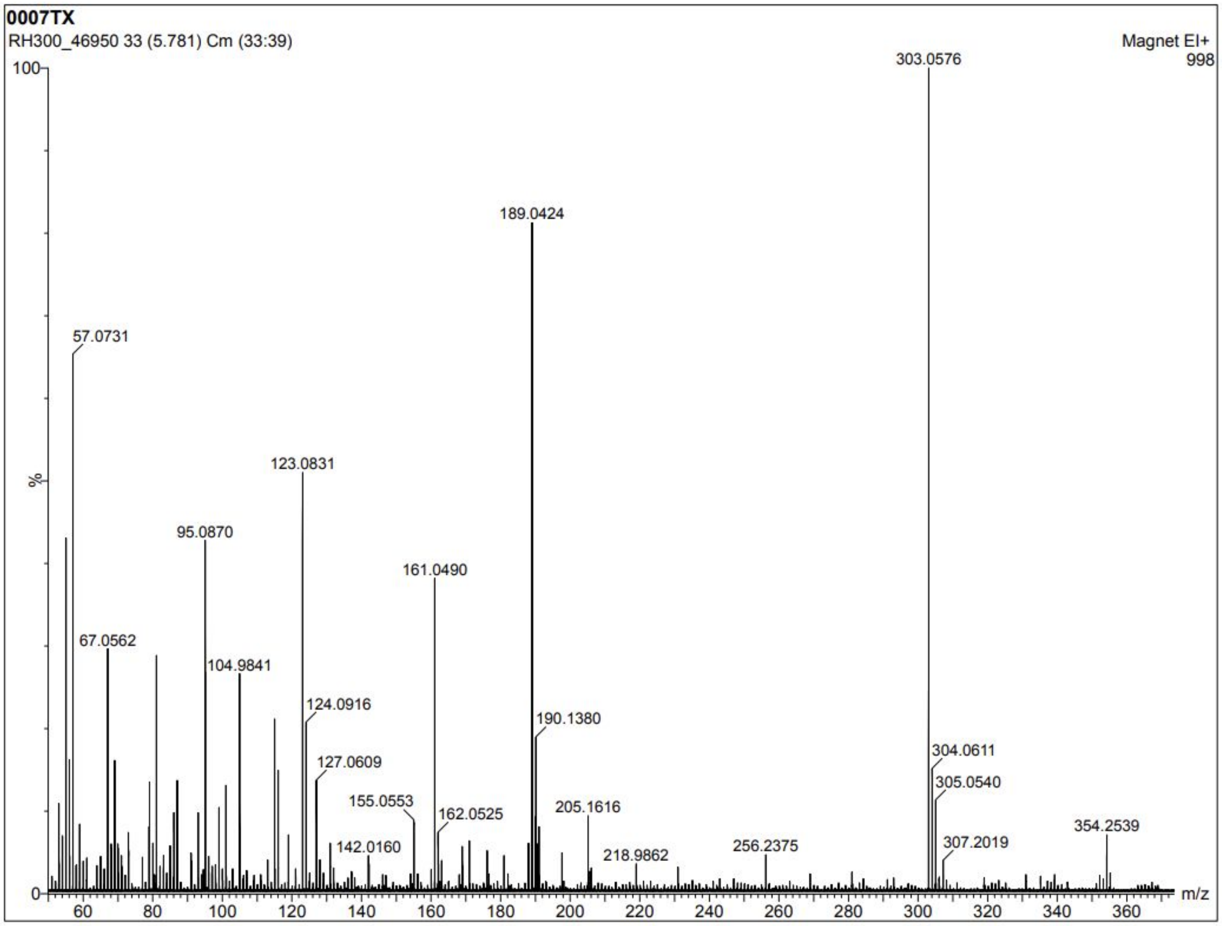

Figure S6. Mass Spectrum of NHS-Lipoic Acid. Calc. for $C_{12}H_{17}NO_4S_2+H^+$ [$M+H^+$]: 304.06, found 304.0611, calc. for $C_{12}H_{17}NO_4S_2+2H^+$ [$M+2H^+$]: 305.06, found 305.0576.

Fourier Transform Infrared Spectroscopy

A

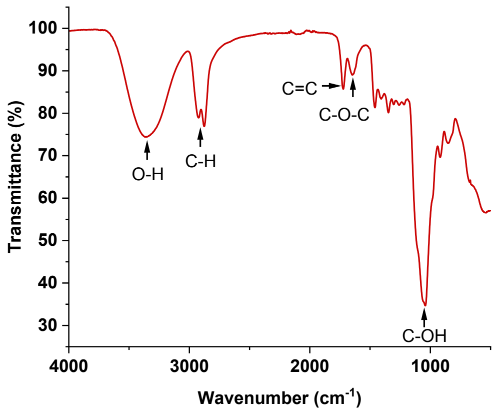

LPG-AGE

B

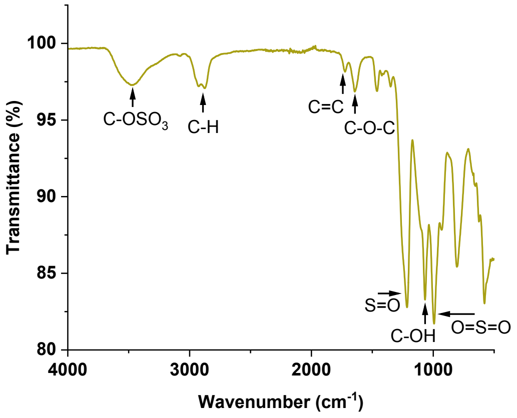

LPGS-AGE

C

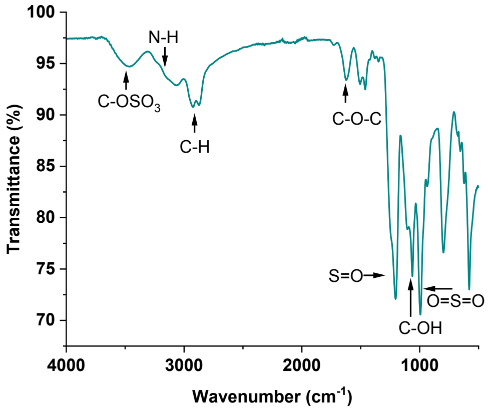

LPGS-CA

D

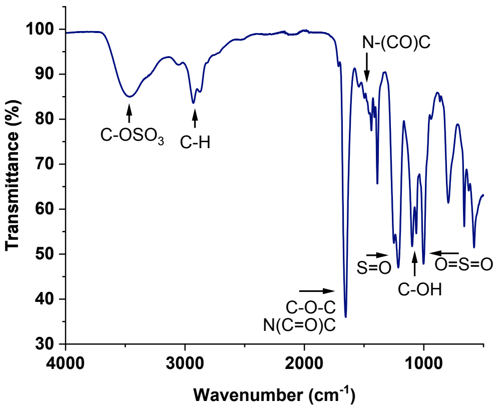

LPGS-LA

Figure S7. FT-IR Spectra of synthetic progress for DDS_1 A) LPG-AGE B)LPGS-AGE, C) LPGS-CA, D) LPGS-LA.

A

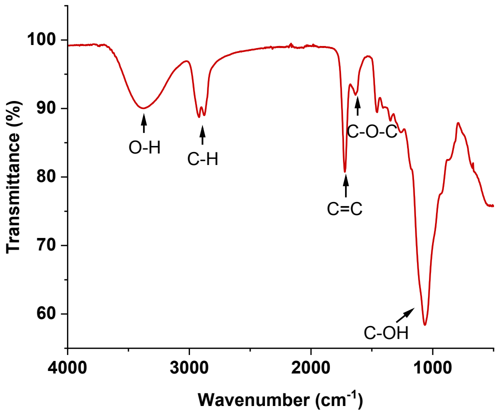

LPG-AGE

B

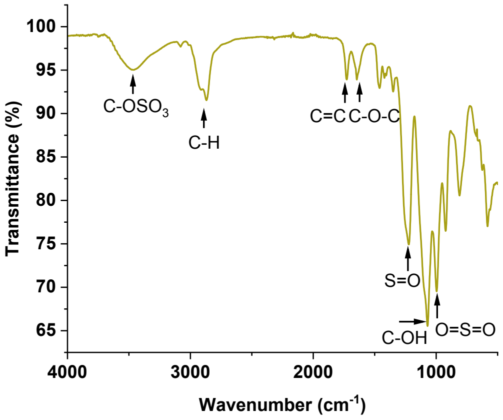

LPGS-AGE

C

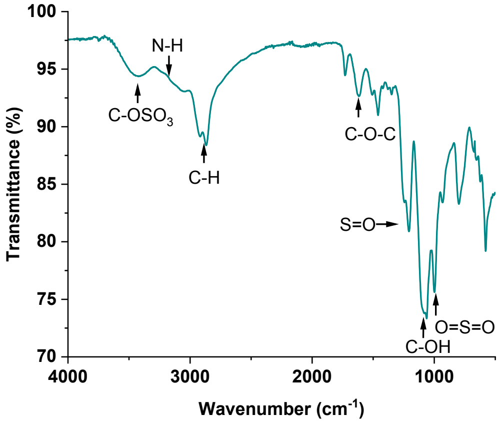

LPGS-CA

D

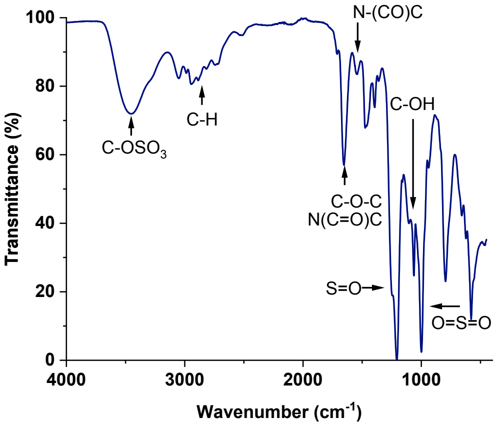

LPGS-LA

Figure S8. FT-IR Spectra of synthetic progress for DDS_2. A) LPG-AGE B) LPGS-AGE, C) LPGS-CA, D) LPGS-LA.

A

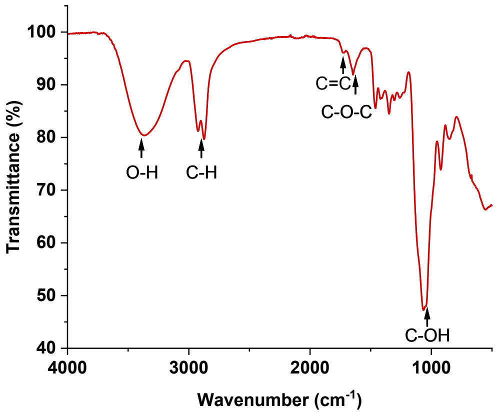

LPG-AGE

B

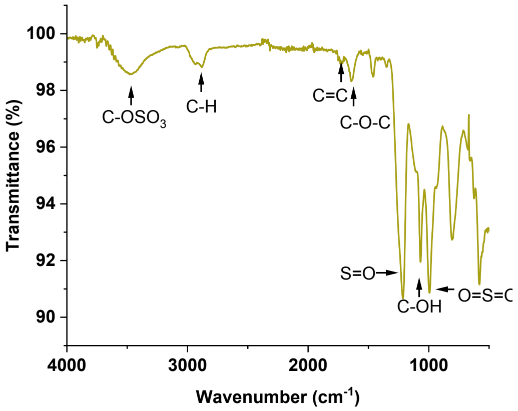

LPGS-AGE

C

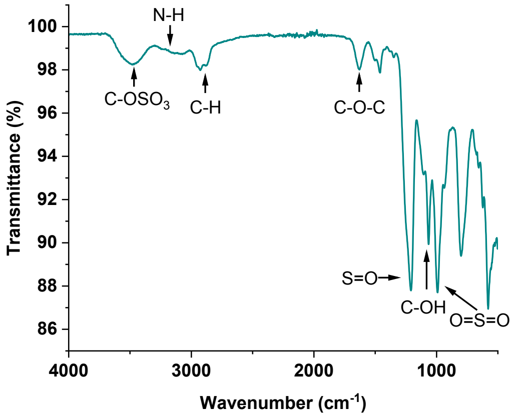

LPGS-CA

D

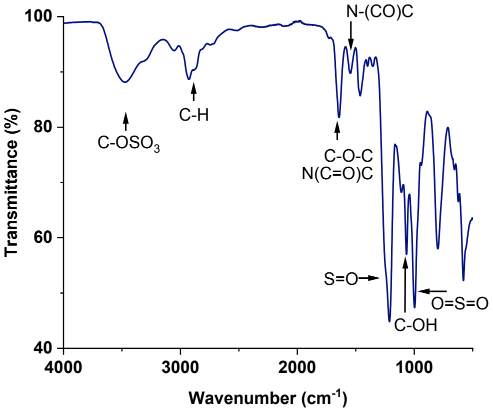

LPGS-LA

Figure S9. FT-IR Spectra of synthetic progress for DDS_3. A) LPG-AGE B) LPGS-AGE, C) LPGS-CA, D) LPGS-LA.

A

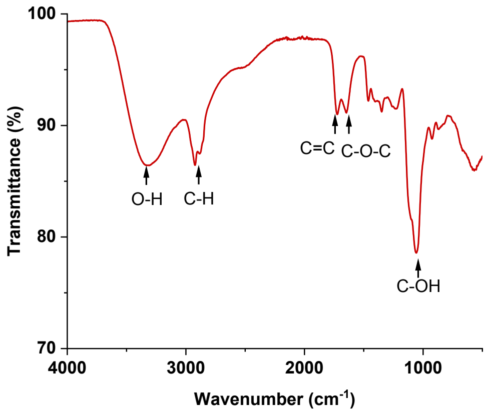

LPG-AGE

B

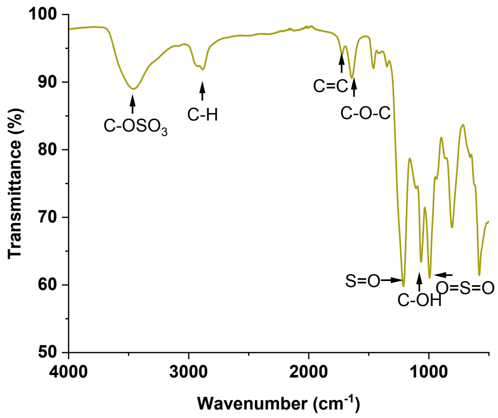

LPGS-AGE

C

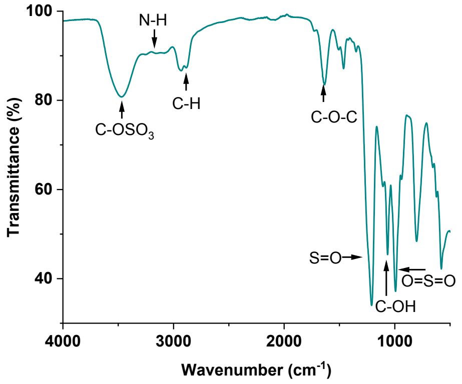

LPGS-CA

D

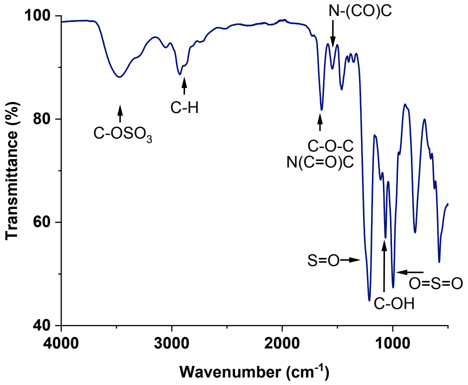

LPGS-LA

Figure S10. FT-IR Spectra of synthetic progress for $LPGS_{40}$-b-LA_{60}, DDS_4 precursor, A) LPG-AGE B)LPGS-AGE, C) LPGS-CA, D) LPGS-LA.

In all FTIR spectra, initial spectra of LPG-AGE indicate stretches of functional groups as expected in the polymer: alcohol groups (3330 cm^{-1}), alkene (2900 cm^{-1}), alkyne (2280 cm^{-1}), ether (1650 cm^{-1}), and primary alcohol (1060 cm^{-1}) stretches. The polymers were then sulfated to LPGS-AGE, where the alcohol signal is largely consumed to indicate C–S stretches (3470 cm^{-1}). Two new signals appear additionally in the fingerprint region (1215 cm^{-1}, 990 cm^{-1}), with a reduction of the signal at 1060 cm^{-1}. Upon the thiol-ene click reaction, alkene stretches from the AGE disappear, and a broader signal of primary amines appears (3150 cm^{-1}). Finally, formation of the lipoic acid amide is evidenced by the strengthening of the amide C=O stretch at 1640 cm^{-1}, and the appearance of N–C stretches at 1540 cm^{-1}.

Thermogravimetric Analysis

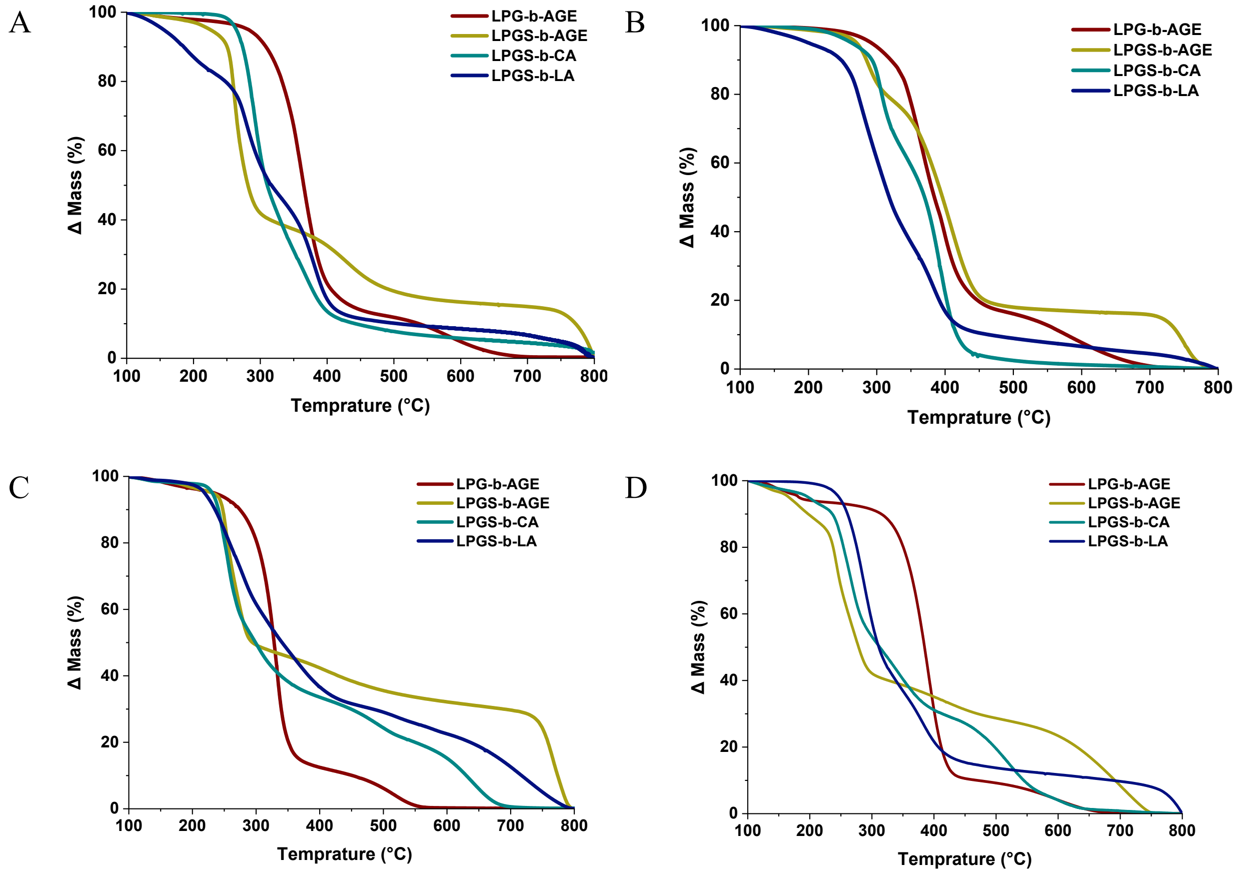

Figure S11. Thermogravimetric Analysis Data for each formed DDS precursor A) DDS_1 B) DDS_2 C) DDS_3 and D) DDS_4.

LPG-PAGE copolymer decomposes between 350 to 420 °C. After sulfation, mass loss is observed at a lower temperature because S–O (240 kJ mol^{-1}) bonds are weaker than C–C (350 kJ mol^{-1}) and C–O (355 kJ mol^{-1}) bonds, which correspond to the degree of sulfation. In DDS_4 (Figure S11 D), 60 % mass loss is observed by 300 °C, corresponding to the $LPG_{40\%}$ converted to $LPGS_{40\%}$. The thiol-ene click reaction displays an additional plateau at 400-500 °C. DDS4 contained 60% AGE. Further modification increased the mass of the repeating unit by approximately 40%, from 114 g mol^{-1} to 191 g mol^{-1}, as confirmed by NMR. This corresponds to full (100%) conversion, which takes into account the addition of cysteamine. Therefore, the mass contribution from the CA units is estimated to be around 60%. Finally, the formation of lipoic acid amide, characterized by stronger amide bonds, explains the higher thermal stability of the final material.

Zeta Surface Potential

Table S1. Zeta surface potential of each synthetic step for respective DDSs.

	DDS_1	DDS_2	DDS_3	DDS_4
**LPG-B-PAGE**	0.0 ± 1.2	-4.0 ± 0.2	2.3 ± 0.3	37.2 ± 0.4
**LPGS-B-PAGE**	-34.0 ± 0.8	-21.6 ± 0.1	-30.2 ± 1.2	-47.1 ± 3.4
**LPGS-B-CA**	-26.6 ± 2.6	20.6 ± 2.1	-33.8 ± 0.3	-23.9 ± 0.8
**LPGS-B-LA**	-34.3 ± 0.9	-35.0 ± 0.7	-20.8 ± 2.8	-29.1 ± 0.5

GPC

Table S2. GPC data for DDSs.

DDS		M_N	M_W	PDI
DDS_1	**LPG-b-PAGE**	4.3	5.7	1.3
	**LPGS-b-PAGE**	4.0	10.2	2.5
DDS_2	**LPG-b-PAGE**	3.9	4.8	1.2
	**LPGS-b-PAGE**	6.2	10.6	1.7
DDS_3	**LPG-b-PAGE**	13.1	15.6	1.2
	**LPGS-b-PAGE**	6.0	17.9	3.0
DDS_4	**LPG-b-PAGE**	7.3	10.2	1.4
	**LPGS-b-PAGE**	7.5	14.7	2.0

Elemental Analysis

Table S3. Elemental Analysis of each DDS and its precursors.

Compound	Element	LPG-b-AGE	LPGS-b-AGE	LPGS-b-CA	LPGS-b-LA
DDS_1	C	54.6	34.8	34.1	28.1
	H	7.1	4.0	5.1	6.9
	N	0.0	0.0	2.9	7.1
	S	0.0	10.3	17.3	10.4
DDS_2	C	60.2	41.1	43.5	41.4
	H	7.2	5.1	6.1	7.0
	N	0.0	0.4	3.4	4.5
	S	0.0	5.9	6.1	15.3
DDS_3	C	49.2	25.6	26.7	28.4
	H	6.8	3.6	4.1	4.5
	N	0.0	0.1	1.4	1.1
	S	0.0	13.6	16.5	18.2
DDS_4	C	52.8	54.7	47.3	41.6
	H	9.0	0.3	7.1	5.4
	N	0.0	0.3	4.7	6.2
	S	0.0	12.8	19.7	13.1

Critical Aggregation Constant

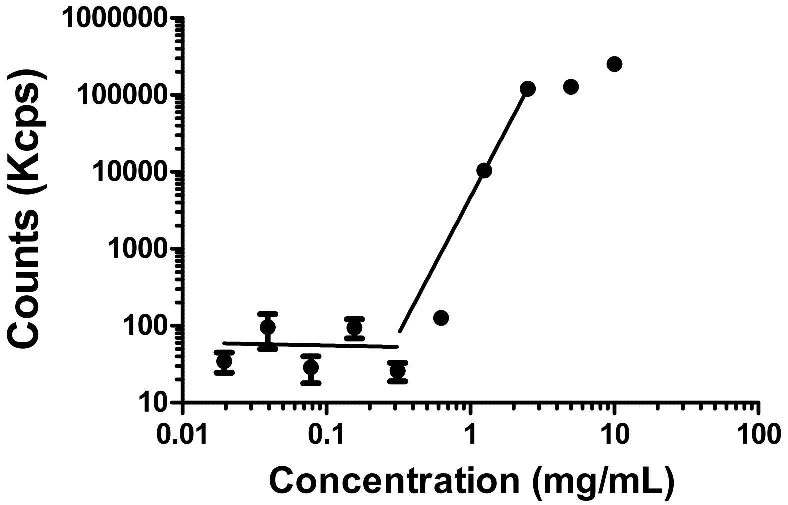

Figure S12. Critical aggregation concentration (CAC) was determined by DLS of $LPGS_{40}$-b-LA_{60} (DDS_4 prior to photocrosslinking) upon serial dilution in DI water measured by count. CAC determined 0.28 ± 0.08 mg/ml.

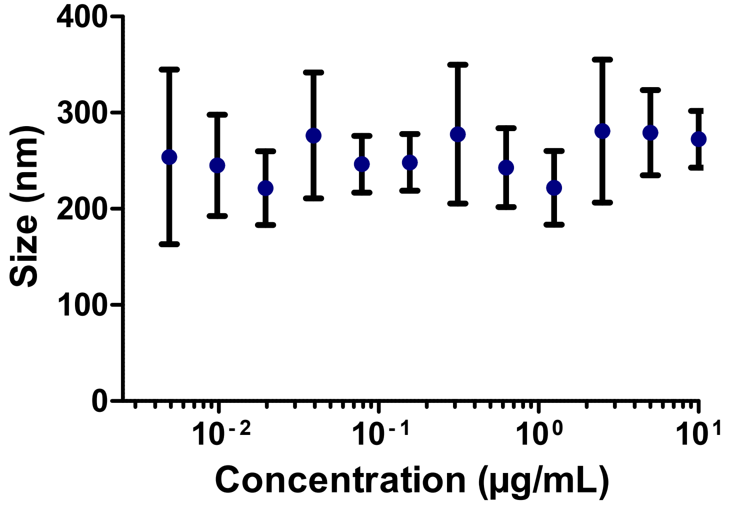

Figure S13. Average size of dissolved DDS_4 determined by DLS over a dilution series in deionized water. Note that detection limit of the machine is 1000 nm and larger aggregations in the form of sheets cannot be measured.

Photocrosslinking

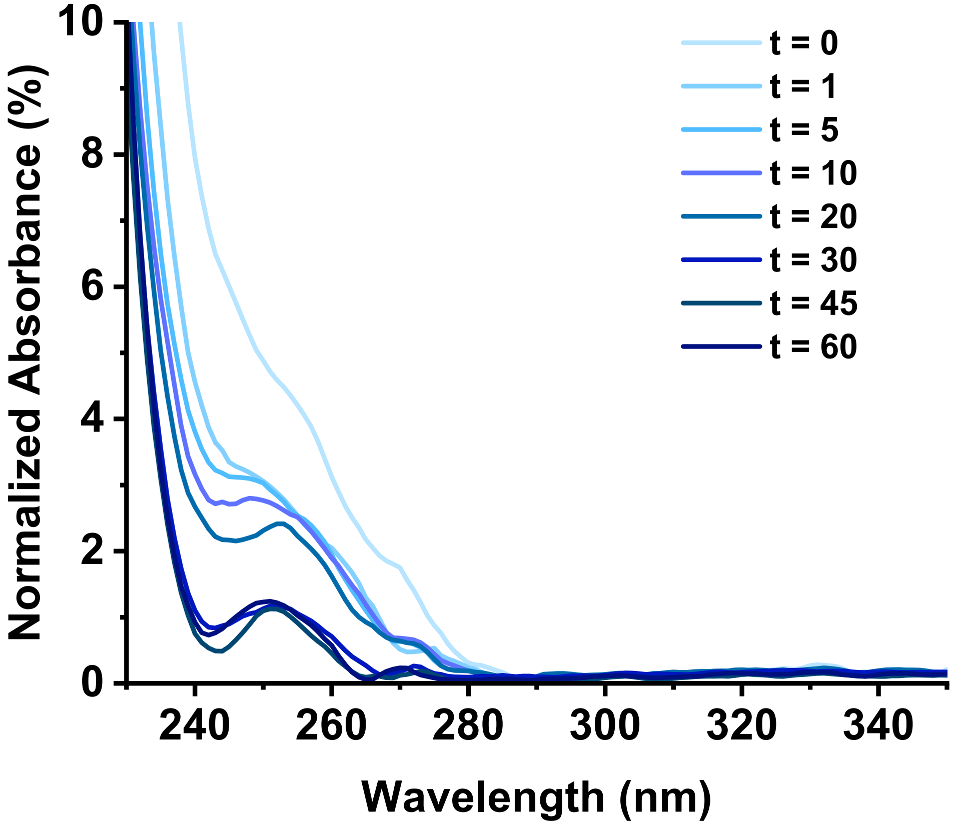

Figure S14. UV-Vis spectra of the conversion from amphiphile ($LPGS_{40}$-b-LA_{60}) to formed DDS nanoparticle (DDS_4) at 1.0 mg/mL in PBS at different time intervals in minutes of irradiation at 370 nm (all spectra normalized at 210 nm and 350 nm).

Scanning Electron Microscope Images

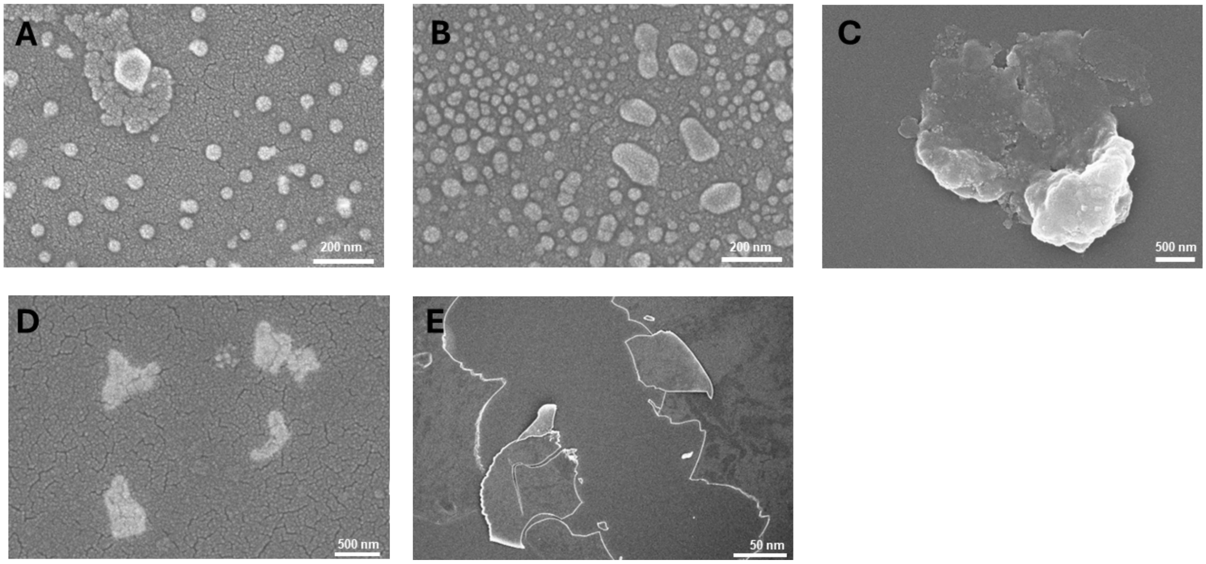

Figure S15. SEM Images of DDS4. Samples correspond to different concentrations prior to dropcasting, gold coating, and imaging A) 1.0 ng/mL, B) 10 ng/mL, C) 0.1 µg/mL, D) 10 µg/mL, E) 0.1 mg/mL

Cryo-Electron Tomography

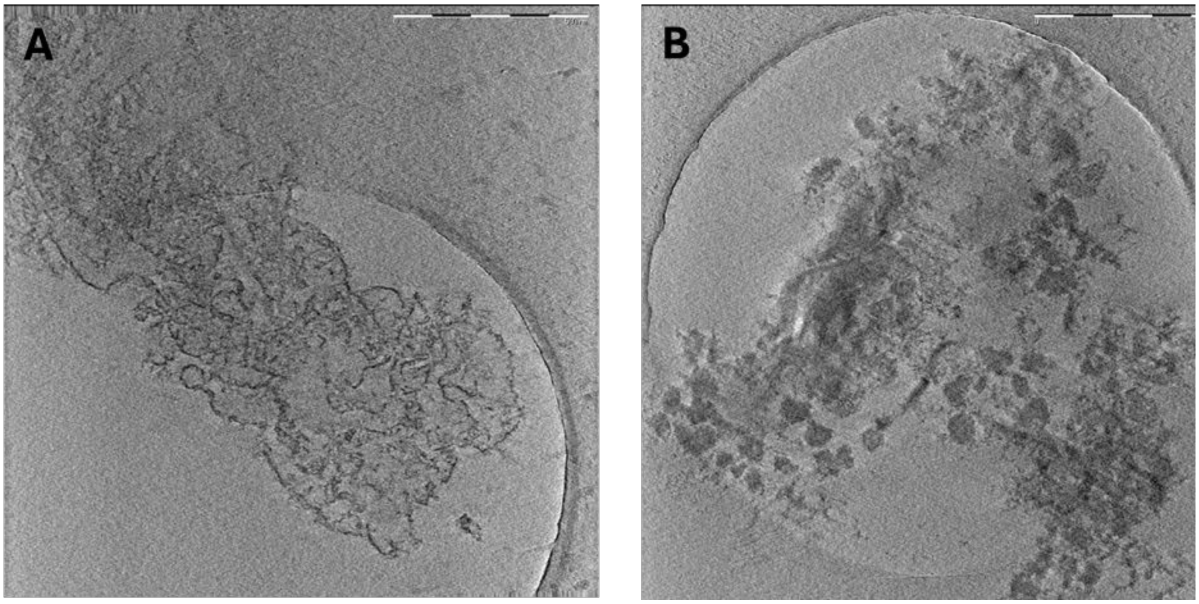

Figure S16. 2D slices through 3D volumes, reconstructed from tomographic tilt series (±64°, 2° increment) of DDS_4, embedded in amorphous ice at 5.0 mg/mL

Release Data

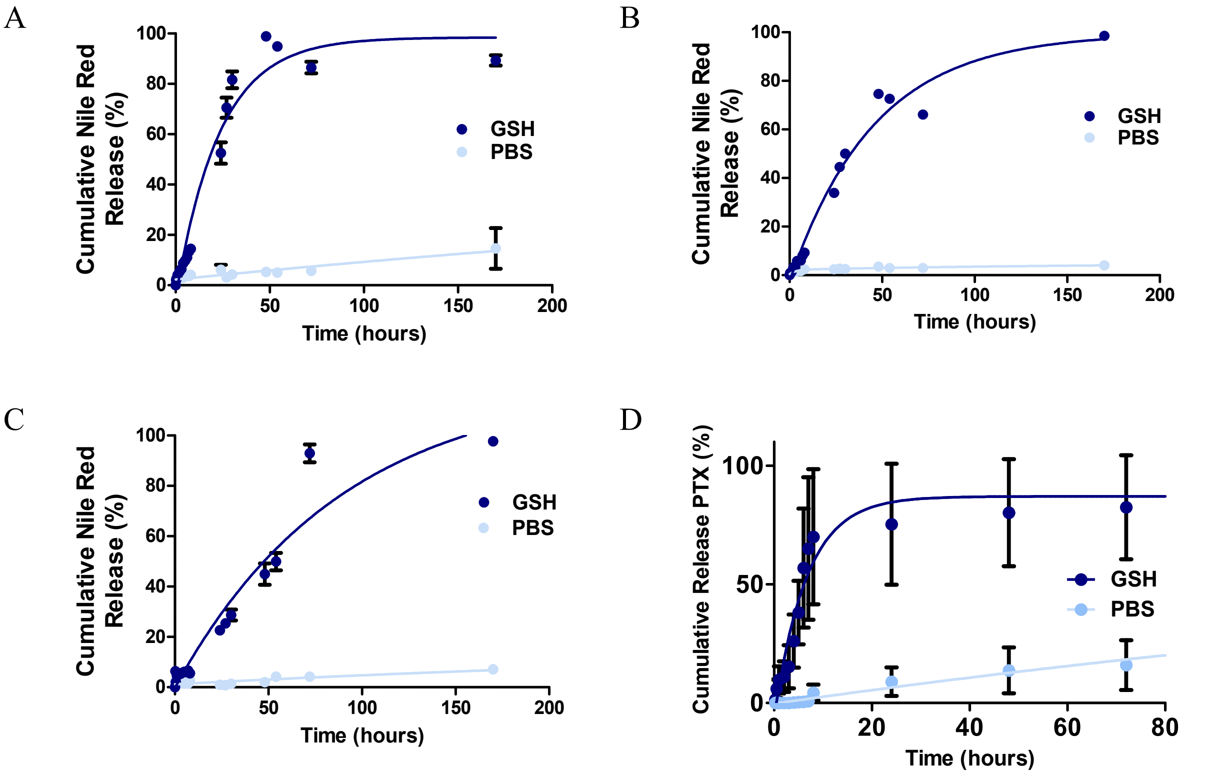

Figure S17. A-D) Cumulative release of Nile Red for each DDS A) DDS_1, B) DDS_2, C) DDS_3, in PBS 7.4 pH and PBS 10mM GSH 7.4 pH over 7 days. The release was calibrated to the highest recorded absorbance value for NR at 268 nm. D) Paclitaxel loaded DDS_4 release over time tracked by HPLC. The data was fitted to a two-phase decay model using GraphPad Prism.

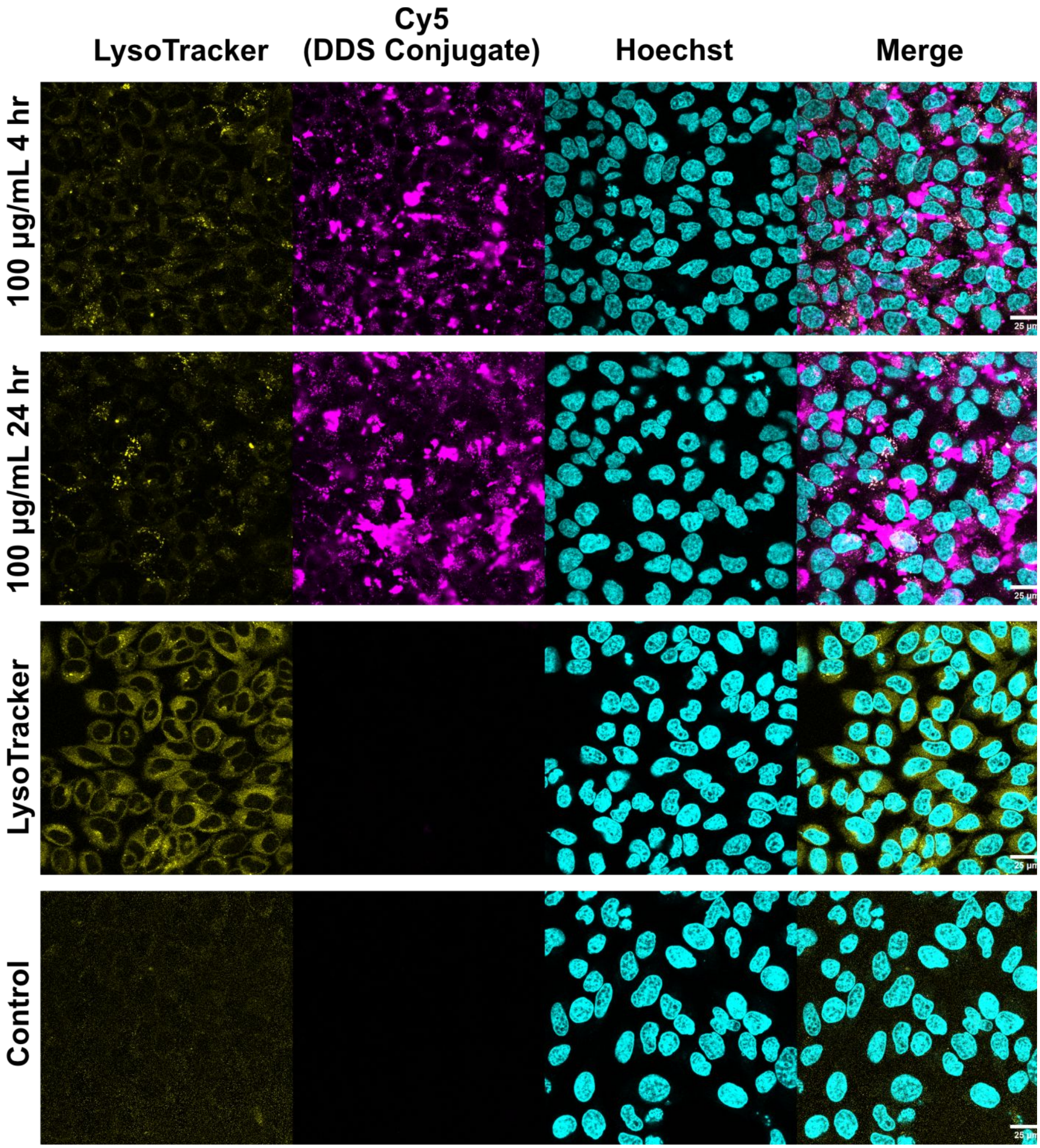

Figure S18. Confocal laser scanning microscope images of HeLa cells cultured with 100 µg/mL of DDS_4 at 4 and 24 hours with a covalently bound maleimide modified Cy5 dye and LysoTracker. Cy5 is shown in magenta, Lysotracker shown in yellow, and the nuclei stained with Hoechst 44432 in cyan. Scale bar is 25 µm.

Cy5 Dye Preparation

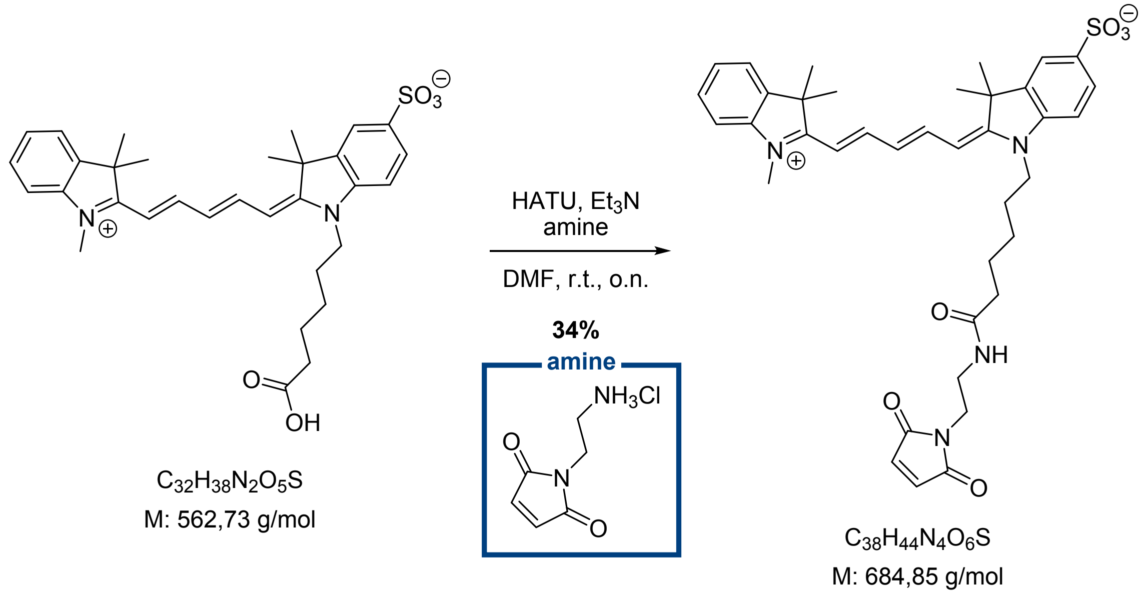

Figure S19. Cy5-Maleimide synthesis from Cy5.

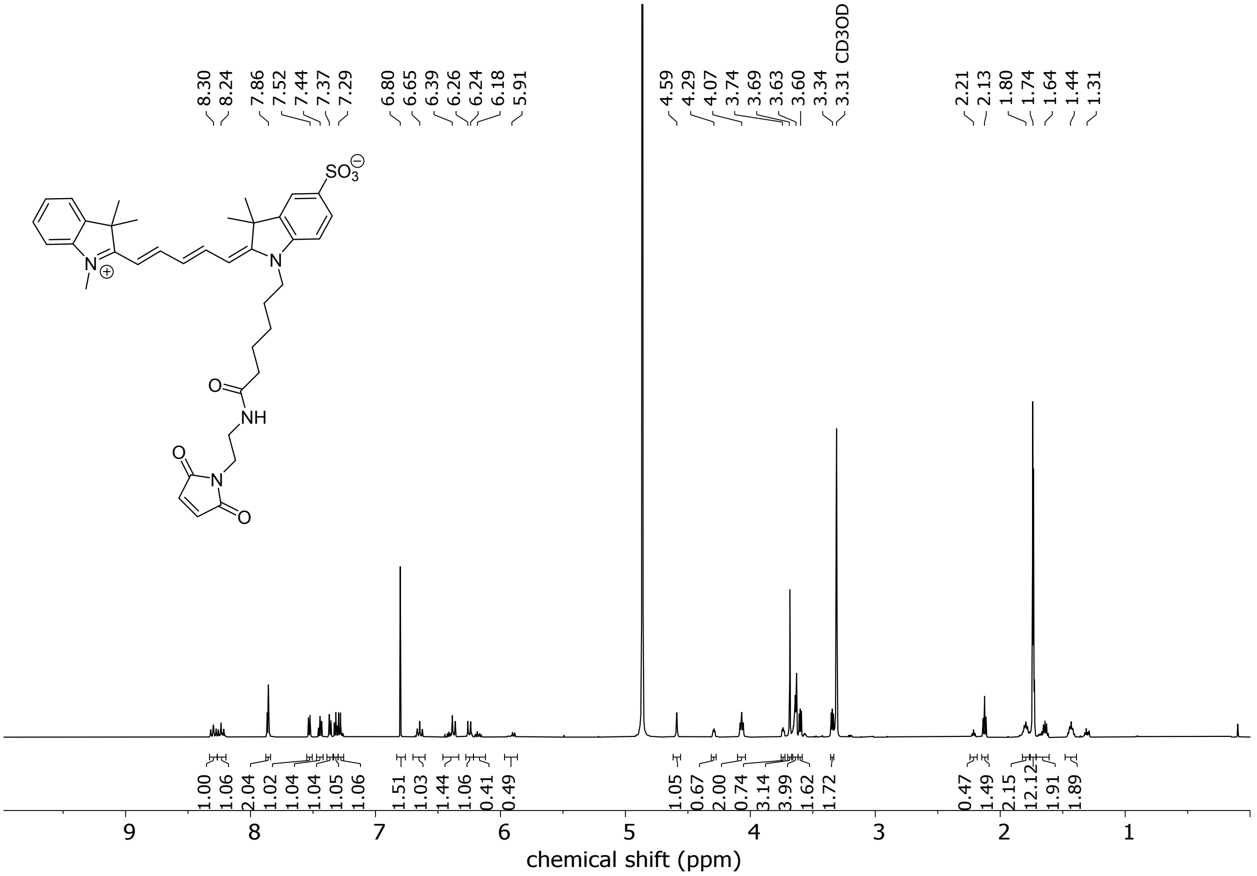

Figure S20. ^{1}H NMR of Cy5-Maleimide in deuterated methanol. δ = 8.35 – 8.25 (m, 1H), 8.23 (t, *J* = 13.1, 1H, major), 8.23 (t, *J* = 13.0 Hz, 1H, minor), 7.87 – 7.84 (m, 2H), 7.53 (dd, *J* = 7.5, 1.2 Hz, 1H), 7.44 (td, *J* = 7.7, 1.2 Hz, 1H), 7.39 – 7.34 (m, 1H), 7.32 (td, J = 7.4, 0.9 Hz, 1H), 7.30 – 7.25 (m, 1H), 6.80 (s, 2H, major), 6.66 (t, *J* = 12.4 Hz, 1H, minor), 6.65 (t, *J* = 12.4 Hz, 1H, major), 6.46 – 6.35 (m, 2H, major), 6.28 – 6.22 (m, 1H), 6.21 – 6.12 (m, 1H, minor), 5.98 – 5.81 (m, 1H, minor), 4.59 (s, br, 1H), 4.33 – 4.24 (m, 2H, minor), 4.07 (t, *J* = 7.5 Hz, 2H), 3.75 – 3.72 (m, 2H, minor), 3.69 (s, 3H, minor), 3.68 (s, 3H, major), 3.66 – 3.62 (m, 4H), 3.61 – 3.57 (m, 2H, major), 3.36 – 3.33 (m, 2H, major), 2.21 (t, *J* = 7.3 Hz, 2H, minor), 2.13 (t, *J* = 7.3 Hz, 2H, major), 1.86 – 1.76 (m, 2H), 1.75 – 1.72 (m, 12H), 1.72 – 1.60 (m, 2H), 1.48 – 1.39 (m, 2H) ppm.

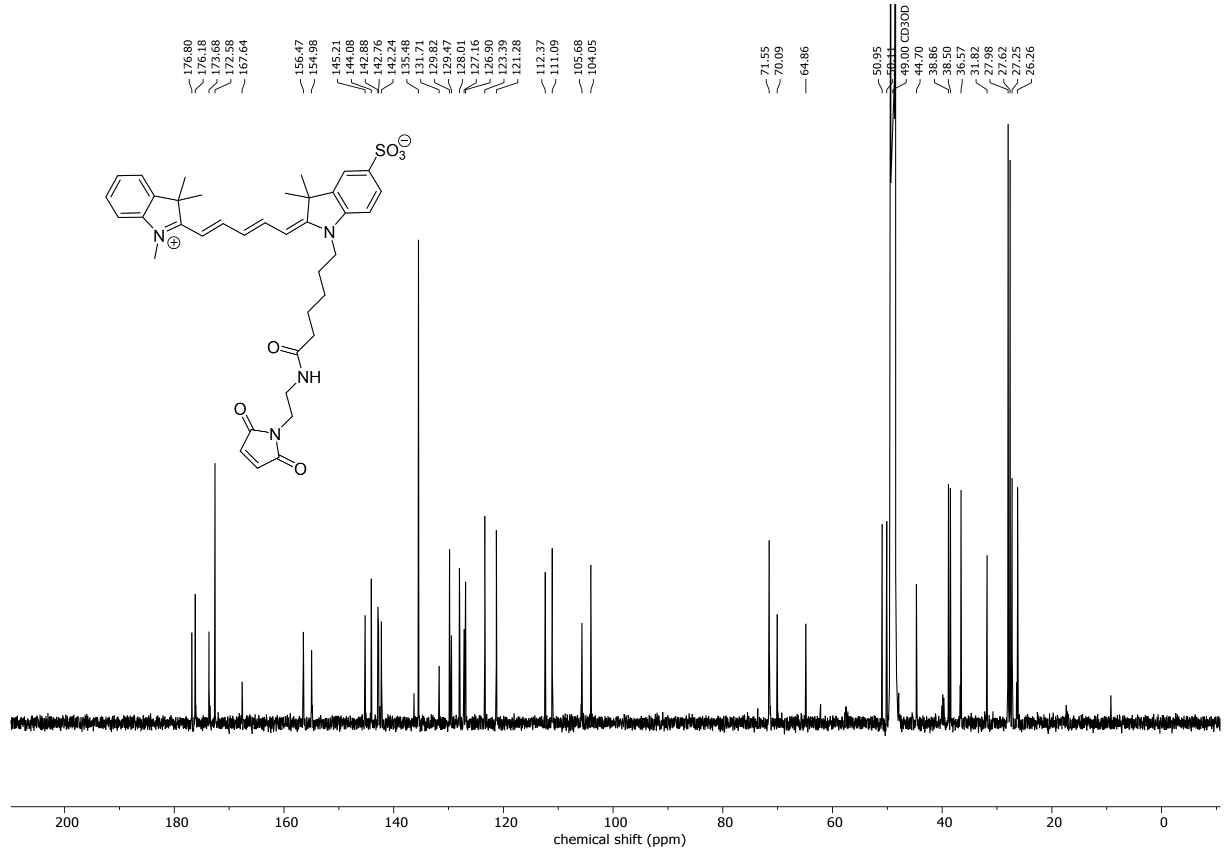

Figure S21. ^{13}C NMR of Cy5-Maleimide in deuterated methanol. ^{13}C NMR (151 MHz, CD_3OD, only major peaks reported): δ = 176.8, 176.1, 173.6, 172.5, 167.6, 156.4, 154.9, 145.2, 144.0, 142.8, 142.7, 142.2, 135.4, 131.7, 129.8, 129.4, 128.0, 127.1, 126.9, 123.3, 121.2, 112.3, 111.0, 105.6, 104.0, 71.5, 70.0, 64.8, 50.9, 50.1, 44.7, 38.8, 38.5, 36.5, 31.8, 27.9, 27.6, 27.2, 26.2 ppm.

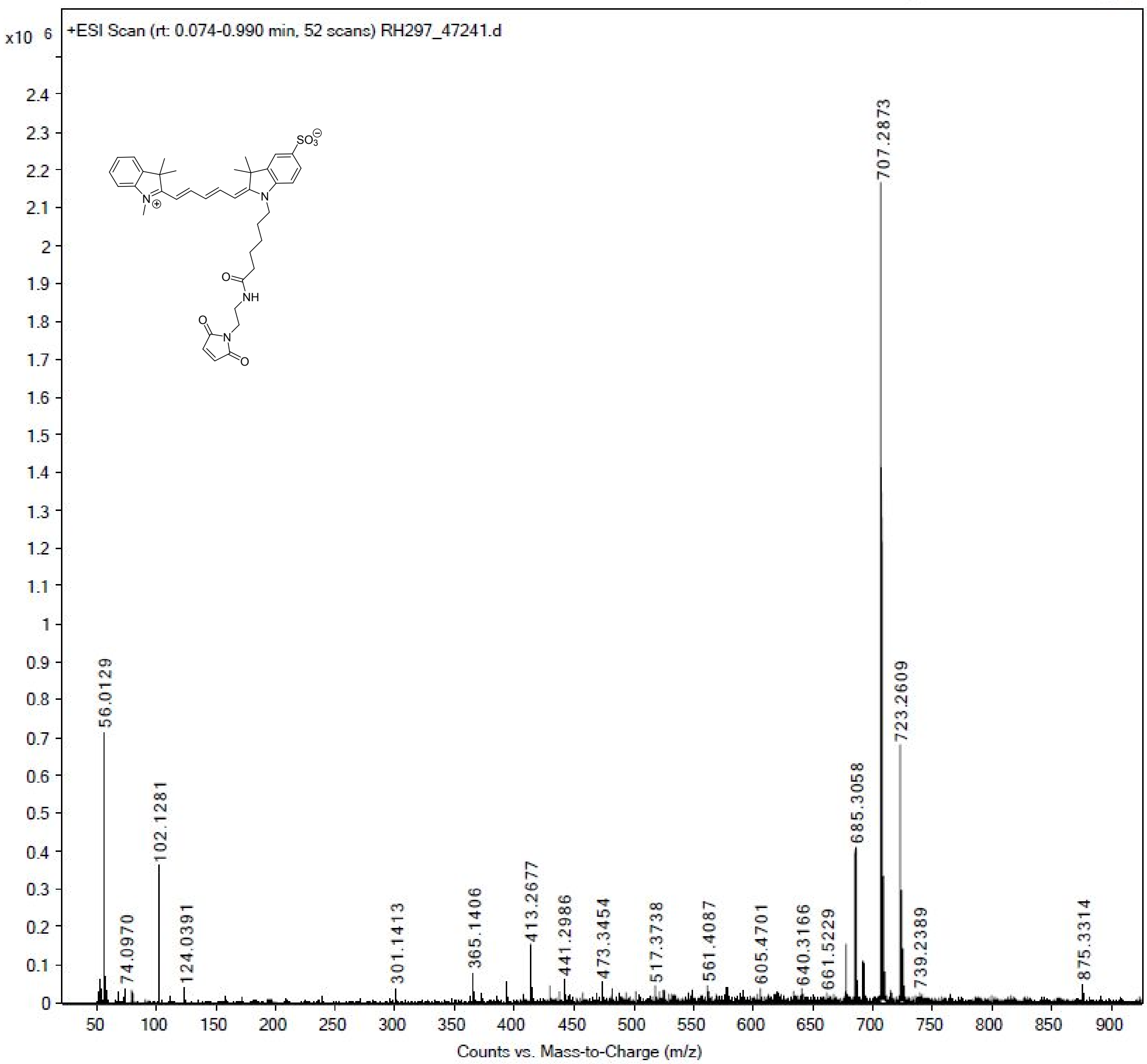

Figure S22. Mass spectrum of Cy5-Maleimide. HRMS (ESI, pos.): *m/z* calc. for $C_{38}H_{44}N_4O_6S+H^+$ [M+H$^+$]: 685.3055, found 685.3058; calc. for $C_{38}H_{44}N_4O_6S+Na^+$ [M + Na$^+$]: 707.2874, found 707.2873.
